# Region-based analysis of rare genomic variants in whole-genome sequencing datasets reveal two novel Alzheimer’s disease-associated genes: *DTNB* and *DLG2*

**DOI:** 10.1038/s41380-022-01475-0

**Published:** 2022-03-04

**Authors:** Dmitry Prokopenko, Sanghun Lee, Julian Hecker, Kristina Mullin, Sarah Morgan, Yuriko Katsumata, Alzheimer’s Disease Neuroimaging Initiative (ADNI), Michael W. Weiner, David W. Fardo, Nan Laird, Lars Bertram, Winston Hide, Christoph Lange, Rudolph E. Tanzi

**Affiliations:** 1 Genetics and Aging Research Unit and The Henry and Allison McCance Center for Brain Health, Department of Neurology, Massachusetts General Hospital, Boston, MA USA; 2 Harvard Medical School, Boston, MA USA; 3 Department of Medical Consilience, Graduate School, Dankook University, Yongin, South Korea; 4 Department of Biostatistics, Harvard T.H. Chan School of Public Health, Boston, MA USA; 5 Channing Division of Network Medicine, Brigham and Women’s Hospital, Boston, MA USA; 6 Department of Pathology, Beth Israel Deaconess Medical Center, 330 Brookline Avenue, Boston, MA USA; 7 Department of Biostatistics, University of Kentucky, Lexington, KY USA; 8 Sanders-Brown Center on Aging, University of Kentucky, Lexington, KY USA; 9 Department of Radiology and Biomedical Imaging, University of California San Francisco, San Francisco, CA USA; 10 Lübeck Interdisciplinary Platform for Genome Analytics, University of Lübeck, Lübeck, Germany; 11 Department of Psychology, University of Oslo, Oslo, Norway

**Keywords:** Genetics, Neuroscience

## Abstract

Alzheimer’s disease (AD) is a genetically complex disease for which nearly 40 loci have now been identified via genome-wide association studies (GWAS). We attempted to identify groups of rare variants (alternate allele frequency <0.01) associated with AD in a region-based, whole-genome sequencing (WGS) association study (rvGWAS) of two independent AD family datasets (NIMH/NIA; 2247 individuals; 605 families). Employing a sliding window approach across the genome, we identified several regions that achieved association *p* values <10^−6^, using the burden test or the SKAT statistic. The genomic region around the dystobrevin beta (*DTNB*) gene was identified with the burden and SKAT test and replicated in case/control samples from the ADSP study reaching genome-wide significance after meta-analysis (*p*_meta_ = 4.74 × 10^−8^). SKAT analysis also revealed region-based association around the Discs large homolog 2 (*DLG2*) gene and replicated in case/control samples from the ADSP study (*p*_meta_ = 1 × 10^−6^). In conclusion, in a region-based rvGWAS of AD we identified two novel AD genes, *DLG2* and *DTNB*, based on association with rare variants.

## Introduction

Alzheimer’s disease (AD) is a heterogeneous, genetically complex neurodegenerative disorder [1]. Over the past 15 years, around 120 genome-wide association studies (GWAS) have been performed to elucidate the genetic architecture underlying AD according to the GWAS catalog [2]. The latest GWAS which has utilized over 1 million individuals ascertained from clinical and proxy-based AD cases and controls has identified 38 independent loci to be associated with AD [3]. GWAS heritability which is tagged by common variants is estimated to be 24-33% [4, 5] - less than a half of the heritability calculated from twin studies [1]. Identification of rare variants associated with AD may help explain the missing heritability, and lead to new biological insights [6]. Several rare variant loci previously associated with AD [7], including *TREM2* [8, 9], have been identified with whole-exome sequencing (WES) studies [10].

Identification of association signals that are driven by rare variants remains cumbersome due to low power and relatively small sample sizes. Hence, aggregation methods, such as burden tests [11, 12] and variance component tests (SKAT) [13, 14], have been developed to jointly test regions of rare variants for association. Combining variant data increases the association signal and reduces the number of statistical tests. While burden tests are most powerful for signals with consistent effect directions, SKAT is more powerful for signals with different effect directions or when the fraction of causal variants within a region is small. Previously, aggregated gene-based association analyses have been successful in identifying exome-wide significant associations with sporadic AD [15–18]. This includes burden rare variant signals in genes with variants previously associated with AD, such as *ABCA7*, *PILRA*, *SORL1*, *TREM2*, as well as novel genes, such as *ZNF655*. Recently, we have performed a rare variant region-based analysis in whole-genome sequencing (WGS) data [19] using a family-based design and a burden family-based association test (FBAT), which was based on estimating the correlation between rare variants based on the observed empirical distribution [20]. Furthermore, an applicable SKAT approach in FBAT was not available at the time of the first manuscript [19]. In the current study, we utilize two novel and complementary region tests (burden and variance component (SKAT)) within a recently developed general framework for exact region-based association testing in family-based designs [21]. The former relates to an improved haplotype algorithm for rare variants recently developed by Hecker et al [22, 23]. This approach alleviates the need to use approximations of the correlation between rare variants: The joint conditional distribution of the rare variants can now be simply obtained by the haplotype approach. Using the proposed region-based testing framework and a systematic region definition, we performed a rvGWAS combining two AD family-based cohorts (605 families; 1509 affecteds; 738 unaffecteds) focusing on rare variants. For replication, we used case/control subjects from NIA ADSP, which included WGS data from a Non-Hispanic White (NHW) subcohort (983 cases; 686 controls), an African-American (AA) subcohort (450 cases; 501 controls), and a Hispanic (HISP) subcohort (486 cases; 613 controls).

Using a *p* value cutoff of 5 × 10^−6^, the burden test and SKAT identified several genomic regions showing association with AD risk. A region identified by the burden test in the *DTNB* gene (*p* = 7 × 10^−8^) was replicated in the NHW samples. SKAT analysis revealed an association with variants encompassing a region around *DLG2* (*p* = 4 × 10^−6^), which replicated in the NHW and the AA samples.

## Methods

### Study populations

#### Discovery family-based dataset

Our discovery dataset consisted of two WGS family-based cohorts: the National Institute of Mental Health (NIMH) family AD cohort [24] and families from the National Institute of Aging Alzheimer’s Disease Sequencing Project [25] (NIA ADSP). Whole-genome sequencing and variant calling in NIMH are described elsewhere [26]. Variant calls for the families from the NIA ADSP cohort were obtained from the National Institute on Aging Genetics of Alzheimer’s Disease Data Storage Site (NIAGADS; URLs) under accession number: NG00067. Both cohorts consisted of multiplex AD families with affected and unaffected siblings (Supplementary table 1). A subject was considered to be affected if he/she was included in one of the following categories: “Definite AD”,”Probable AD” or”Possible AD”. Subjects were counted as unaffected by AD if they either had no diagnosis of dementia, or suspected dementia (46 subjects), or non-AD dementia (10 subjects). Given FBAT’s robustness to model misspecifications [27] (including case vs. control status) this strategy will generally result in an increase of statistical power (owing to the larger sample size). It is important to note that even in families where the non-AD or suspected dementia is actually the result of an overlooked diagnosis of AD, this will not lead to spurious findings but merely reduce statistical power. It is important to note that NIA ADSP families by design did not include individuals with two APOE-ε4 alleles. After standard quality control, both cohorts were merged together.

#### NIA ADSP case-control dataset

WGS variant calls for the NIA ADSP replication case-control dataset were obtained from the NIAGADS under accession number: NG00067 and consisted of the ADSP Discovery-Extension Case-Control WGS dataset [25] and the ADNI Case-Control WGS dataset. Samples were remapped to GRCh38 and jointly called with the families from the NIA ADSP cohort. Full details can be found on NIAGADS (https://dss.niagads.org/datasets/ng00067/) and elsewhere [28]. Briefly, a subject was considered affected, if he/she met the NINCDS-ADRDA criteria for possible, probable, or definite AD, had documented age at onset or age at death (for pathologically verified cases), and *APOE* genotyping. All controls were 60 or more years old and were free of dementia.

### Quality control

Briefly, we have excluded individuals based on genotyping rate, inbreeding coefficient, and family mismatches using identity by descent (IBD) sharing coefficients. After sample-based quality control, we have combined two WGS family-based cohorts: NIMH (1393 individuals in 446 families) and families from NIA ADSP (854 individuals in 159 families). In the merged dataset we excluded multiallelic variants, monomorphic variants, singletons (i.e., variants with only one alternate allele across the dataset and variants seen only in one family), indels, and variants which had one missing allele among 2 alleles in an individual. The remaining variants were filtered based on Mendel errors, genotyping rate (95%), Hardy–Weinberg equilibrium (*p* < 1e−08), calling quality in TOPMed (URLs), which is a large WGS database with >100,000 individuals sequenced jointly, and alternate allele frequency as defined in gnomAD (AF ≤ 1% in either whole gnomAD or nonFinnish European sample) (URLs).

### WGS regional-based analysis

We have performed a whole-genome scan for our combined family-based AD dataset using a newly developed exact framework in FBAT for region-based association testing [21]. We grouped rare variants in nonoverlapping consecutive sets of ten based on our discovery family-based dataset. For each set of rare variants, we considered the burden test and the SKAT test using Affection Status (coded as 0/1 for unaffected/affected) minus offset as phenotype. We selected an offset of 0.15 which approximately corresponds to the population prevalence of AD. We have used FBAT [29] (URLs), R [30], snakemake [31] and bash commands to implement and run the described analyses.

### Replication

Replication significance level was set to 0.05. In addition, we compute the combined meta *p* values and highlight regions that reached overall Bonferroni-corrected genome-wide significance (*p* < 6.24 × 10^−8^). We have used the SKAT package to perform Burden and SKAT-O tests on the same sets of rare variants in the case-control replication cohorts. We chose SKAT-O because it is the optimal test in an extended family of SKAT tests and combines the power of a burden and SKAT test and it implements a small-sample adjustment procedure (*n* < 2000) [14]. As covariates, we used sequencing center, age, sex, and principal components (to account for population structure). To recover more recent admixture and better correct for population stratification in WGS data we calculated principal components based on 100,000 rare variants using the Jaccard index [32]. Those rare variants were randomly selected from a pruned subset of rare variants (*R*^2^ < = 0.01). We have also performed meta-analysis among datasets with similar ethnical background using the Fisher’s combined probability test.

### RNA-Seq and microarray analysis

We explored *DLG2* and *DTNB* genes’ expression based on the Human Protein Atlas (HPA) RNA-seq data (URLs) and tested for differential expression of synaptic and immune-related genes including *DLG2* and *DTNB* genes between normal controls (*N* = 173, aged 20–99 years) and AD cases (*N* = 80) in the brain regions including hippocampus, entorhinal cortex, superior frontal cortex, and post-central gyrus using microarray dataset GSE48350, which is available from the Gene Expression Omnibus Web site (URLs). Differential expression was tested using the “GEO2R” tool.

### Network construction

We used Cytoscape 3.8.0 and the StringDB protein-protein interaction resource [33] (URLs) using only identified protein-protein interactions. Using a background that agglomerates protein-protein interaction datasets, we seeded the network with *DLG2* and *DTNB* and identified direct associations between proteins and *DLG2* and *DTNB* in a global network. Results were combined using the Genemania server (Utilizing significantly co-expressed genes across several experimental datasets) [34] to further capture functional relationships and to build a combined protein-protein/gene co-expression network.

### Functional enrichment

Functional enrichment within the network was performed using the remote StringDB server linked to Cytoscape “String App Enrichment function” [35], producing enrichments using the hypergeometric test, with *P* values corrected for multiple testing using the method of Benjamini and Hochberg in known molecular pathways and GO terms as described in Franceschini et al. [36].

## Results

In a region-based whole-genome sequencing rvGWAS focusing on rare genomic variants, we combined two AD family-based cohorts, the NIMH Alzheimer’s disease genetics initiative study (NIMH) and the family component of the NIA ADSP sample. The combined sample consisted of 1509 affected and 738 unaffected siblings in families of predominantly European ancestry (Supplementary Table 1, Methods). 8,011,126 variants passed strict quality control and alternate allele frequency (AF) filter of ≤1% (based on gnomAD [37]). We grouped rare variants into consecutive non-overlapping regions/windows of ten variants and performed a rare variant WGS scan over the whole genome (801,124 windows). We employed a recently developed framework for exact regional-based analysis within FBAT [21] to analyze these sets of rare variants using both the burden test and SKAT. These tests are able to detect different configurations of disease regions - dense regions with the same effect directions (burden test) or less dense signals with varying effect directions (SKAT).

**Fig. 1:**
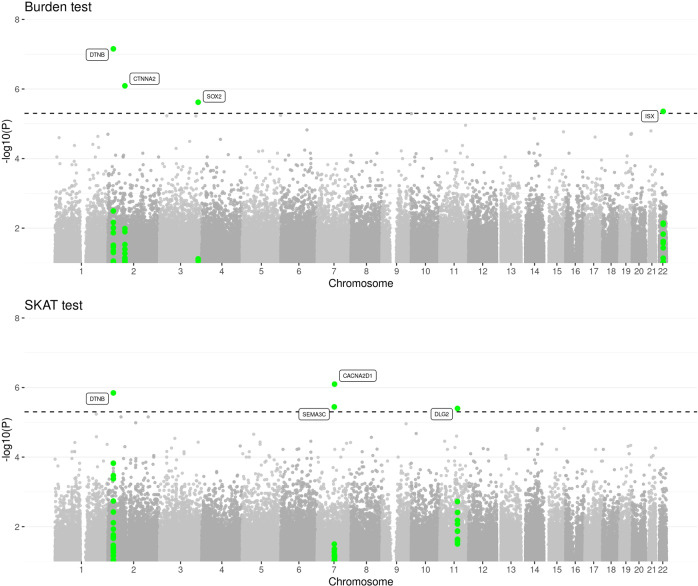
Manhattan plots of sets of rare variants in the whole-genome scan of the family-based discovery dataset using the burden and SKAT test. Dashed line corresponds to suggestive threshold of 5 × 10^−6^.

Since we restricted our analysis to rare variants (i.e., AF < 0.01) and given our modest sample size in the family-discovery cohort, we have used a relatively liberal *p* value threshold *p* < 5 × 10^−6^ to identify “suggestive associations” by burden test or SKAT. A stricter Bonferroni-corrected significance threshold would be *p* = 6.24 × 10^−8^. Seven loci exhibited suggestive evidence for association with AD risk (Fig. 1, Supplementary Fig. 1, Table 1). For replication analysis, we selected the unrelated, multiethnic WGS AD subset from the NIA ADSP dataset (Methods). This dataset consists of three subpopulations: NHW (*n* = 1669), AA (*n* = 951), HISP (*n* = 1099) (Sample sizes after quality control; Supplementary Table 1). A region located downstream to *DTNB*, with a burden *p* value of 7 × 10^−8^ and a SKAT *p* value of 1.4 × 10^−6^ in the discovery dataset, showed a burden *p* value of 0.0324 and a SKAT-O *p* value of 0.054 in the replication ADSP NHW dataset (Table 1 and Supplementary Table 2). Another region, located in an intron of *DLG2* with a SKAT *p* value of 4 × 10^−6^ in the discovery family-based dataset, showed replication with a significant *p* value of 0.0143 in the ADSP NHW dataset and a *p* value of 0.053 in the ADSP AA dataset (Table 1 and Supplementary Table 3). Two other regions showed nominally significant replication *p* values in AA (*SEMA3C*, *p* = 0.046) and HISP (*ISX*, *p* = 0.014), but not in NHW.

**Table 1:** Top regions based on the burden or SKAT test with *p* ≤ 5e−06 in the discovery family-based dataset using whole-genome scan.

First SNV in the region (chr:pos:ref:alt)	Last SNV in the region (chr:pos:ref:alt)	Nearest protein-coding gene	Discovery dataset (NIMH + NIA families)		Replication dataset NHW ADSP		Meta-analysis of family-based discovery and NHW ADSP replication datasets		Replication dataset AA ADSP		Replication dataset HISP ADSP	
			*P* value	Number of simulations	*P* value	Number of SNVs in the region used in the test	Fisher chi-squared test statistic	*P* value	*P* value	Number of SNVs in the region used in the test	*P* value	Number of SNVs in the region used in the test
Burden test												
2:25703040:G:A	2:25707419:T:G	*DTNB*	7.00E−08	1.00E + 08	0.032	5	39.808	4.74E−08	0.390	9	0.353	8
2:79854141:T:G	2:79856252:C:T	*CTNNA2*	8.10E−07	1.00E + 08	0.799	6	28.500	9.88E−06	0.774	8	0.785	7
3:181942653:A:G	3:181946475:G:A	*SOX2*	2.40E−06	1.00E + 07	0.317	6	28.177	1.15E−05	0.619	9	0.163	8
22:35048628:G:C	22:35053269:C:T	*ISX*	4.40E−06	1.00E + 07	0.766	8	25.201	4.58E−05	0.827	9	0.014	10
Variance component test												
11:83498255:A:G	11:83500398:T:G	*DLG2*	4E−06	1.00E + 07	0.014	5	33.352	1E−06	0.053	8	0.893	9
2:25703040:G:A	2:25707419:T:G	*DTNB*	1.4E−06	1.00E + 08	0.054	5	32.737	1.4E−06	0.409	9	0.455	8
7:82268137:T:C	7:82271095:A:T	*CACNA2D1*	8E−07	1.00E + 07	0.591	5	29.131	7.4E−06	0.278	8	0.090	8
7:81141368:T:G	7:81143780:C:T	*SEMA3C*	3.6E−06	1.00E + 07	0.251	5	27.832	1.4E−05	0.046	9	0.694	10

*SNV* single nucleotide variant, *chr* chromosome, *pos* position according to GRCh38, *ref* reference allele, *alt* alternate allele, *NHW* non Hispanic white, *AA* African-American, *HISP* Hispanic.

During the peer review of this paper NIA ADSP released a larger AD WGS case-control dataset with a 4.5-fold sample size increase for the NHW subpopulation, which we used for additional replication analyses. To this end, we recalculated the burden and the SKAT-O test statistics for our two top hits in *n* = 7413 NHW individuals (previously *n* = 1669). The analyses revealed only minor changes with respect to our original replication results, i.e., the burden *p* value for *DTNB* increased to 0.0698 while the SKAT-O *p* value for *DLG2* decreased to 0.0038.

Both *DLG2* and *DTNB* are highly expressed in the brain based on RNA-data from three different sources: Internally generated Human Protein Atlas (HPA) RNA-seq data, RNA-seq data from the Genotype-Tissue Expression (GTEx) project, and CAGE data from the FANTOM5 project, as well as the consensus dataset for each gene derived from the Human Protein Atlas [38] (Supplementary Figs. 2, 3). It is important to mention that besides being expressed in different brain tissues, *DTNB* is also highly expressed in salivary gland tissue, which might be due to the fact that the resulting protein, β-dystrobrevin, is only found in non-muscle tissues [39]. In the Alzheimer’s Disease Dataset analysis [40] (GSE48350) from the GEO database [41] expression of *DLG2* and *DTNB* is significantly decreased in AD compared to control subjects in at least one of two microarray ids corresponding to the genes (Supplementary Table 4).

Network analysis revealed a network of 33 proteins interacting with DLG2 and DTNB that were enriched for neuronal synaptic functions (Supplementary Fig. 4). Functional enrichment of the subnetwork of proteins directly interacting with DLG2 and DTNB revealed 694 enriched GO process/ pathway terms (Supplementary table 5). The most enriched part of the network was for proteins interacting with DLG2 that are connected to neurexins and neuroligins, as well as trafficking of AMPA receptors. DLG2 also interacted with 4 proteins (NOS1, ERBB4, DLGAP2, NRXN3) which were among the top 1000 leading AD-associated single rare variants and regions [19], and 4 proteins (GRIN1, GRIN2A, GRIN2B, GAPDH) associated with AD in the KEGG Alzheimer’s pathway. DLG2 and DTNB also share protein-protein or co-expression interactions through KIF1B, MLC1, and SH3D19.

## Discussion

Here, we describe a comprehensive region-based analysis of Alzheimer’s disease using WGS datasets. We specifically searched for novel AD association signals driven by regions of rare variants in a large family-based cohort. To account for different disease region specifications, we employed both the burden test and SKAT. This yielded seven regions of suggestive evidence (*p* < 5 × 10^−6^) for association with AD risk in the family datasets. These results were followed up with replication analysis in independent case-control samples of different ethnicities. Two loci, *DTNB* and *DLG2*, showed consistent evidence of replication in the NHW subpopulation. The *DLG2* region was also confirmed in the African-American sample.

*DLG2* encodes a member of the membrane-associated guanylate kinase family, also known as post-synaptic density protein, PSD-93. Down-regulation of synaptic scaffolding proteins, including DLG2, has been described as an early event in AD [42]. *DLG2* has been proposed as a potential target for AD based on an integrated metabolomics-genetics-imaging systems approach in Agora (URLs); agonism of *DLG2* is predicted to reduce disease progression. An expression dataset of AD in the GEO database revealed reduced expression of *DLG2* in AD versus controls. A common variant in *DLG2*, rs683250, was previously associated with increases of shape asymmetry in controls as compared to individuals with dementia [43]. This same variant is in linkage disequilibrium (LD, D’ = 1) with all rare variants of the *DLG2* region found to be associated with AD here. *DLG2* variant, rs286043 (AF = 0.03), which exhibited suggestive evidence for association with AD risk in IGAP (*p* = 5e−06), is in LD with 4 out of 10 variants from our *DLG2* AD-associated region, suggesting possible allelic heterogeneity. *DLG2* has previously been associated with schizophrenia [44] and autism [45, 46]. Along these lines, *DLG2* deficiency in mice has been reported to lead to reduced sociability and increased repetitive behavior along with aberrant synaptic transmission in the dorsal striatum [47].

β-Dystrobrevin (*DTNB*) is associated with neurons in the cortex, hippocampus, and cerebellum, as well as other brain regions, implying that it might be an important protein involved in some neuronal pathways in the brain [39], and has also been reported to be enriched in the post-synaptic density (PSD), a protein complex associated with postsynaptic membranes of excitatory synapses [48, 49]. Kinesin superfamily motor proteins (KIF) are responsible for anterograde protein transport within the axon of various cellular cargoes, including synaptic and structural proteins [50]. Dysregulated KIF expression has also been associated with early AD pathology [51], and β-Dystrobrevin interacts directly with kinesin heavy chain in the brain [52]. Expression of α-Dystrobrevin (*DTNA*), which a paralog of *DTNB*, has been associated with dementia status and P-tau levels in temporal cortex [53]. Dystrobrevin-binding protein 1, also known as dysbindin, has been reported to be associated with schizophrenia [54, 55]. Thus, both novel AD gene candidates identified in this study have been associated with post-synaptic function. They have also shown association with risk for schizophrenia. While schizophrenia and Alzheimer’s disease have, generally, different etiologies (including genetics), other studies have shown that there are some important intersections between both diseases, especially related to post-synaptic density proteins [56–58]. The two novel AD genes identified here might be located at one of such functional intersections.

Family-based designs are completely robust to potential misspecification of disease model and population stratification. This led us to define the family-based portion of our study as our “discovery” dataset. In contrast, the “replication” portion of our study utilized datasets from unrelated cases and controls. Two regions (*DLG2* and *DTNB*) were validated in the replication cohort. Utilizing the increased sample size of the latest NIAGADS release, the replication evidence became stronger for *DLG2* and slightly decreased for *DTNB*.

Concurrent to our analyses, we became aware of an independent WES study of AD cerebrospinal fluid (CSF) biomarker levels by Neumann et al. [59]. Intriguingly, that study also identified rare variants in *DTNB* showing experiment-wide rare-variant association signals with the CSF biomarkers analyzed. Thus, there are now two studies using independent datasets, sequencing techniques and different AD-related outcome phenotypes converging on highly significant rare-variant association signals in *DTNB*, emphasizing the likely crucial – and hitherto unrecognized – role of this gene in AD pathogenesis.

Our approach utilized two region-based tests (burden and SKAT) in a family-based design, in which the joint distribution of rare variants is not estimated, but rather obtained by the haplotype algorithm for FBAT, which is robust against population structure and admixture, and allows for construction of exact or simulation-based *p* values. Previously, we performed region-based rare variant testing, but with different region definitions, and using only burden tests with empirical estimation of the variant correlations and asymptotic *p* values [19]. We also note that by utilizing a window size of 10 consecutive variants, we could have missed sparsely distributed signals. Since the number of possible haplotypes increases exponentially with the number of variants tested, larger window sizes were computationally infeasible.

In summary, we identified two novel loci associated with AD, based on association with rare variants in *DLG2* and *DTNB* in a family-based AD WGS sample using methods that are robust to population structure. Both novel AD genes identified here encode post-synaptic density proteins and have been implicated for roles in schizophrenia. These loci showed replication in an independent AD WGS dataset with unrelated cases and controls and, additionally, *DTNB* was recently highlighted in independent work [59] on the effect of rare-variants on AD CSF biomarker levels. In this separate work Neumann et al. using WES reported rare-variant association signals between *DTNB* and AD CSF biomarker levels in two independent datasets, which makes further studies on the role of *DTNB* in AD pathogenesis warranted.

### URLs

FBAT, https://sites.google.com/view/fbatwebpage; gnomAD, https://gnomad.broadinstitute.org/; Agora AMP-AD, https://agora.ampadportal.org/genes; TOPMED, https://www.nhlbiwgs.org/; Human Protein Atlas, https://www.proteinatlas.org/; GEO database, https://www.ncbi.nlm.nih.gov/geo/; NIAGADS, https://www.niagads.org/; StringDB, https://string-db.org/.

## Supplementary information

Supplementary material

## Data Availability

The NIMH dataset analyzed during the current study is available from the corresponding author on reasonable request. The family component and the case-control component of the NIA ADSP WGS dataset is available from DSS NIAGADS under accession number: NG00067. Data used in preparation of this article were in part obtained from the Alzheimer’s Disease Neuroimaging Initiative (ADNI) database (adni.loni.usc.edu). As such, the investigators within the ADNI contributed to the design and implementation of ADNI and/or provided data but did not participate in analysis or writing of this report.

## Code availability

All scripts used to generate the region-based analyses are available from the authors upon request.
