# Supplementary material for: Region-based analysis of rare genomic variants in whole-genome sequencing datasets reveal two novel Alzheimer’s disease-associated genes: DTNB and DLG2
Source: Mol Psychiatry. 2022 Mar 4;27(4):1963–9. doi: 10.1038/s41380-022-01475-0 (PMC9126808; doi:10.1038/s41380-022-01475-0)
Supplement: Supplementary file 1 — Supplementary material [file 41380_2022_1475_MOESM1_ESM.docx]

# Supplementary Materials: Region-based analysis of rare genomic variants in whole-genome sequencing datasets reveal two novel Alzheimer’s disease-associated genes: DTNB and DLG2

## Supplementary Acknowledgments

The authors would like to thank the staff from the National Institute of Mental Health (NIMH) Divisions of Clinical and Treatment Research (DCTR) and Epidemiology and Services Research (DESR), including David Shore, MD, Mary Farmer, MD, MPH, Debra Wynne, MSW, Steven 0. Moldin, PhD, Darrell G. Kirch, MD (1989-1994), Nancy E. Maestri, PhD (1992-1994), William Huber (1989-1995), Pamela Wexler (1995-), and Darrel A. Regier, MD, MPH. They would also like to thank the study staff at all three sites and the data management staff at SRA Technologies, Inc., particularly Cheryl McDonnell, PhD, for the care and attention that they paid to all aspects of the study. The authors are also extremely grateful to the families whose participation made this work possible.

Data collection and sharing for this project was funded by the Alzheimer's Disease Neuroimaging Initiative (ADNI) (National Institutes of Health Grant U01 AG024904) and DOD ADNI (Department of Defense award number W81XWH-12-2-0012). ADNI is funded by the National Institute on Aging, the National Institute of Biomedical Imaging and Bioengineering, and through generous contributions from the following: AbbVie, Alzheimer’s Association; Alzheimer’s Drug Discovery Foundation; Araclon Biotech; BioClinica, Inc.; Biogen; Bristol-Myers Squibb Company; CereSpir, Inc.;Cogstate;Eisai Inc.; Elan Pharmaceuticals, Inc.; Eli Lilly and Company; EuroImmun; F. Hoffmann-La Roche Ltd and its affiliated company Genentech, Inc.; Fujirebio; GE Healthcare; IXICO Ltd.; Janssen Alzheimer Immunotherapy Research & Development, LLC.; Johnson & Johnson Pharmaceutical Research & Development LLC.; Lumosity; Lundbeck; Merck & Co., Inc.; Meso Scale Diagnostics, LLC.;NeuroRx Research; Neurotrack Technologies;Novartis Pharmaceuticals Corporation; Pfizer Inc.; Piramal Imaging; Servier; Takeda Pharmaceutical Company; and Transition Therapeutics.The Canadian Institutes of Health Research is providing funds to support ADNI clinical sites in Canada. Private sector contributions are facilitated by the Foundation for the National Institutes of Health (www.fnih.org). The grantee organization is the Northern California Institute for Research and Education, and the study is coordinated by the Alzheimer’s Therapeutic Research Institute at the University of Southern California. ADNI data are disseminated by the Laboratory for Neuro Imaging at the University of Southern California. A complete list of acknowledgements for the use of ADNI dataset can be found here: <http://adni.loni.usc.edu/wp-content/uploads/how_to_apply/ADNI_Acknowledgement_List.pdf>.

Data for this study were prepared, archived, and distributed by the National Institute on Aging Alzheimer’s Disease Data Storage Site (NIAGADS) at the University of Pennsylvania (U24-AG041689), funded by the National Institute on Aging.

The Alzheimer’s Disease Sequencing Project (ADSP) is comprised of two Alzheimer’s Disease (AD) genetics consortia and three National Human Genome Research Institute (NHGRI) funded Large Scale Sequencing and Analysis Centers (LSAC). The two AD genetics consortia are the Alzheimer’s Disease Genetics Consortium (ADGC) funded by NIA (U01 AG032984), and the Cohorts for Heart and Aging Research in Genomic Epidemiology (CHARGE) funded by NIA (R01 AG033193), the National Heart, Lung, and Blood Institute (NHLBI), other National Institute of Health (NIH) institutes and other foreign governmental and non-governmental organizations. The Discovery Phase analysis of sequence data is supported through UF1AG047133 (to Drs. Schellenberg, Farrer, Pericak-Vance, Mayeux, and Haines); U01AG049505 to Dr. Seshadri; U01AG049506 to Dr. Boerwinkle; U01AG049507 to Dr. Wijsman; and U01AG049508 to Dr. Goate and the Discovery Extension Phase analysis is supported through U01AG052411 to Dr. Goate, U01AG052410 to Dr. Pericak-Vance and U01 AG052409 to Drs. Seshadri and Fornage. Data generation and harmonization in the Follow-up Phases is supported by U54AG052427 (to Drs. Schellenberg and Wang).

The ADGC cohorts include: Adult Changes in Thought (ACT), the Alzheimer’s Disease Centers (ADC), the Chicago Health and Aging Project (CHAP), the Memory and Aging Project (MAP), Mayo Clinic (MAYO), Mayo Parkinson’s Disease controls, University of Miami, the Multi-Institutional Research in Alzheimer’s Genetic Epidemiology Study (MIRAGE), the National Cell Repository for Alzheimer’s Disease (NCRAD), the National Institute on Aging Late Onset Alzheimer's Disease Family Study (NIA-LOAD), the Religious Orders Study (ROS), the Texas Alzheimer’s Research and Care Consortium (TARC), Vanderbilt University/Case Western Reserve University (VAN/CWRU), the Washington Heights-Inwood Columbia Aging Project (WHICAP) and the Washington University Sequencing Project (WUSP), the Columbia University Hispanic- Estudio Familiar de Influencia Genetica de Alzheimer (EFIGA), the University of Toronto (UT), and Genetic Differences (GD).

The CHARGE cohorts are supported in part by National Heart, Lung, and Blood Institute (NHLBI) infrastructure grant HL105756 (Psaty), RC2HL102419 (Boerwinkle) and the neurology working group is supported by the National Institute on Aging (NIA) R01 grant AG033193. The CHARGE cohorts participating in the ADSP include the following: Austrian Stroke Prevention Study (ASPS), ASPS-Family study, and the Prospective Dementia Registry-Austria (ASPS/PRODEM-Aus), the Atherosclerosis Risk in Communities (ARIC) Study, the Cardiovascular Health Study (CHS), the Erasmus Rucphen Family Study (ERF), the Framingham Heart Study (FHS), and the Rotterdam Study (RS). ASPS is funded by the Austrian Science Fond (FWF) grant number P20545-P05 and P13180 and the Medical University of Graz. The ASPS-Fam is funded by the Austrian Science Fund (FWF) project I904),the EU Joint Programme - Neurodegenerative Disease Research (JPND) in frame of the BRIDGET project (Austria, Ministry of Science) and the Medical University of Graz and the Steiermärkische Krankenanstalten Gesellschaft. PRODEM-Austria is supported by the Austrian Research Promotion agency (FFG) (Project No. 827462) and by the Austrian National Bank (Anniversary Fund, project 15435. ARIC research is carried out as a collaborative study supported by NHLBI contracts (HHSN268201100005C, HHSN268201100006C, HHSN268201100007C, HHSN268201100008C, HHSN268201100009C, HHSN268201100010C, HHSN268201100011C, and HHSN268201100012C). Neurocognitive data in ARIC is collected by U01 2U01HL096812, 2U01HL096814, 2U01HL096899, 2U01HL096902, 2U01HL096917 from the NIH (NHLBI, NINDS, NIA and NIDCD), and with previous brain MRI examinations funded by R01-HL70825 from the NHLBI. CHS research was supported by contracts HHSN268201200036C, HHSN268200800007C, N01HC55222, N01HC85079, N01HC85080, N01HC85081, N01HC85082, N01HC85083, N01HC85086, and grants U01HL080295 and U01HL130114 from the NHLBI with additional contribution from the National Institute of Neurological Disorders and Stroke (NINDS). Additional support was provided by R01AG023629, R01AG15928, and R01AG20098 from the NIA. FHS research is supported by NHLBI contracts N01-HC-25195 and HHSN268201500001I. This study was also supported by additional grants from the NIA (R01s AG054076, AG049607 and AG033040 and NINDS (R01 NS017950). The ERF study as a part of EUROSPAN (European Special Populations Research Network) was supported by European Commission FP6 STRP grant number 018947 (LSHG-CT-2006-01947) and also received funding from the European Community's Seventh Framework Programme (FP7/2007-2013)/grant agreement HEALTH-F4-2007-201413 by the European Commission under the programme "Quality of Life and Management of the Living Resources" of 5th Framework Programme (no. QLG2-CT-2002-01254). High-throughput analysis of the ERF data was supported by a joint grant from the Netherlands Organization for Scientific Research and the Russian Foundation for Basic Research (NWO-RFBR 047.017.043). The Rotterdam Study is funded by Erasmus Medical Center and Erasmus University, Rotterdam, the Netherlands Organization for Health Research and Development (ZonMw), the Research Institute for Diseases in the Elderly (RIDE), the Ministry of Education, Culture and Science, the Ministry for Health, Welfare and Sports, the European Commission (DG XII), and the municipality of Rotterdam. Genetic data sets are also supported by the Netherlands Organization of Scientific Research NWO Investments (175.010.2005.011, 911-03-012), the Genetic Laboratory of the Department of Internal Medicine, Erasmus MC, the Research Institute for Diseases in the Elderly (014-93-015; RIDE2), and the Netherlands Genomics Initiative (NGI)/Netherlands Organization for Scientific Research (NWO) Netherlands Consortium for Healthy Aging (NCHA), project 050-060-810. All studies are grateful to their participants, faculty and staff. The content of these manuscripts is solely the responsibility of the authors and does not necessarily represent the official views of the National Institutes of Health or the U.S. Department of Health and Human Services.

The four LSACs are: the Human Genome Sequencing Center at the Baylor College of Medicine (U54 HG003273), the Broad Institute Genome Center (U54HG003067), The American Genome Center at the Uniformed Services University of the Health Sciences (U01AG057659), and the Washington University Genome Institute (U54HG003079).

Biological samples and associated phenotypic data used in primary data analyses were stored at Study Investigators institutions, and at the National Cell Repository for Alzheimer’s Disease (NCRAD, U24AG021886) at Indiana University funded by NIA. Associated Phenotypic Data used in primary and secondary data analyses were provided by Study Investigators, the NIA funded Alzheimer’s Disease Centers (ADCs), and the National Alzheimer’s Coordinating Center (NACC, U01AG016976) and the National Institute on Aging Genetics of Alzheimer’s Disease Data Storage Site (NIAGADS, U24AG041689) at the University of Pennsylvania, funded by NIA, and at the Database for Genotypes and Phenotypes (dbGaP) funded by NIH. This research was supported in part by the Intramural Research Program of the National Institutes of health, National Library of Medicine. Contributors to the Genetic Analysis Data included Study Investigators on projects that were individually funded by NIA, and other NIH institutes, and by private U.S. organizations, or foreign governmental or nongovernmental organizations.

## Supplementary Figures

*Supplementary Figure 1: Quantile-quantile plots of rare-variant region-based analysis in the whole genome scan of the family-based discovery dataset.*


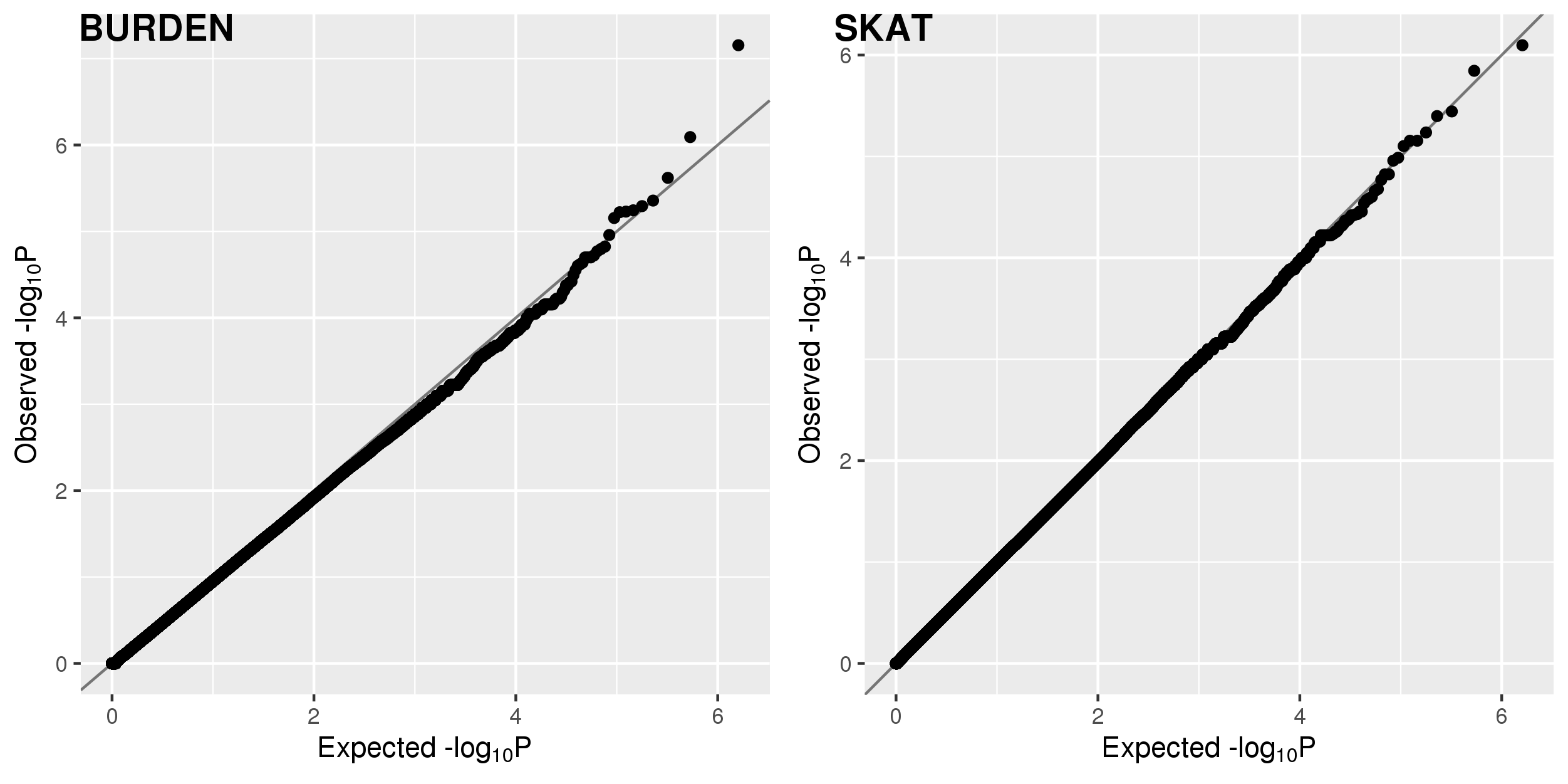


*Supplementary Figure 2: RNA consensus Normalized eXpression (NX) levels for DLG2 in 55 tissue types and 6 blood cell types, created by combining the data from the three transcriptomics datasets (HPA, GTEx and FANTOM5). Color-coding is based on tissue groups, each consisting of tissues with functional features in common. Image generated at Human Protein Atlas: https://www.proteinatlas.org/ENSG00000150672-DLG2/tissue.*


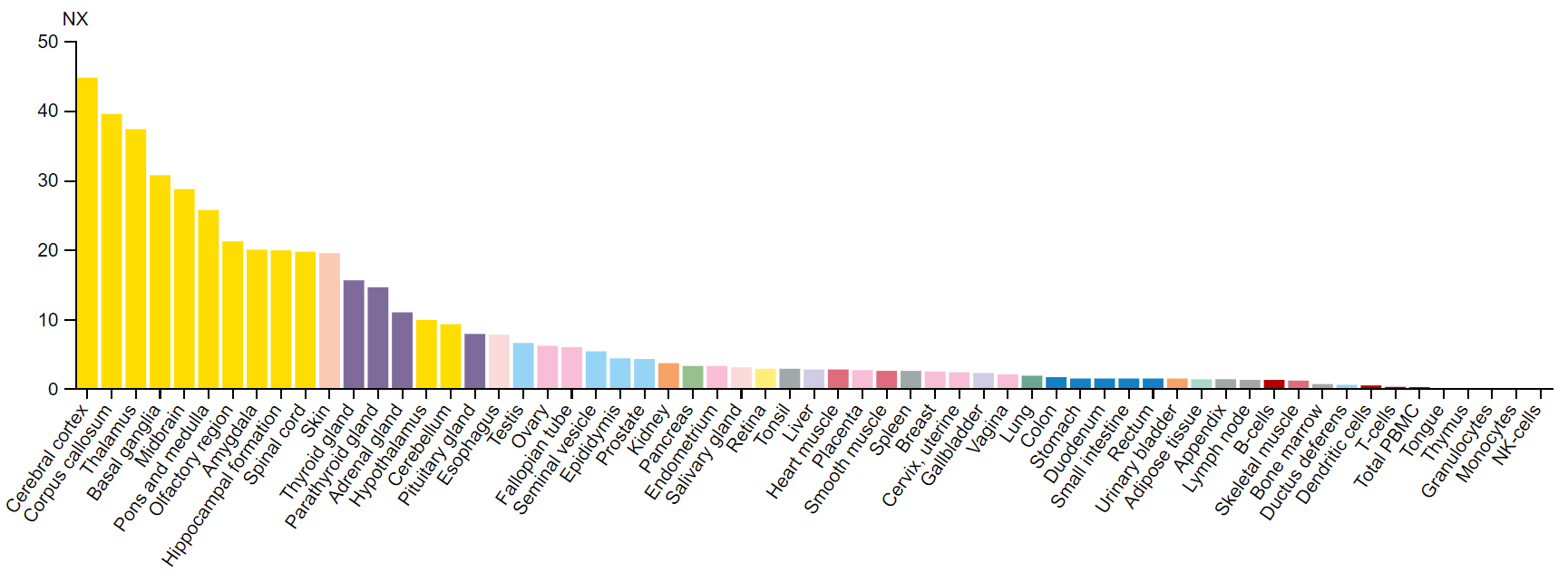


*Supplementary Figure 3: RNA consensus Normalized eXpression (NX) levels for DTNB in 55 tissue types and 6 blood cell types, created by combining the data from the three transcriptomics datasets (HPA, GTEx and FANTOM5). Color-coding is based on tissue groups, each consisting of tissues with functional features in common. Image generated at Human Protein Atlas: https://www.proteinatlas.org/ENSG00000138101-DTNB/tissue.*


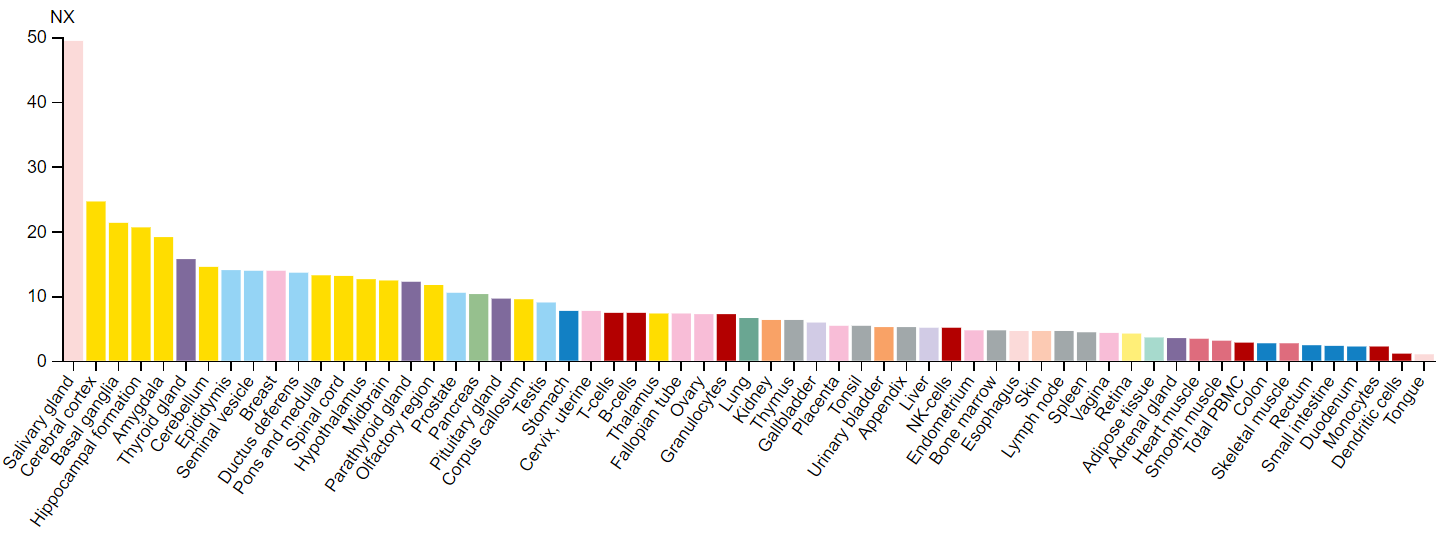


*Supplementary Figure 4: Direct interaction and co-expression of proteins with DLG2 (red border), channel-associated protein of synapse-110 (chapsyn-110), and DTNB (red border), dystrobrevin beta, a component of the dystrophin-associated protein complex. Members of KEGG Alzheimer’s pathway (teal nodes). Top 1000 SNV AD-associated genes identified in Prokopenko et al. (green nodes), and/or top 1000 regions of AD-associated genes (dark blue nodes). DLG2 and DTNB share interactions with KIF1B, MLC1, and SH3D19.*


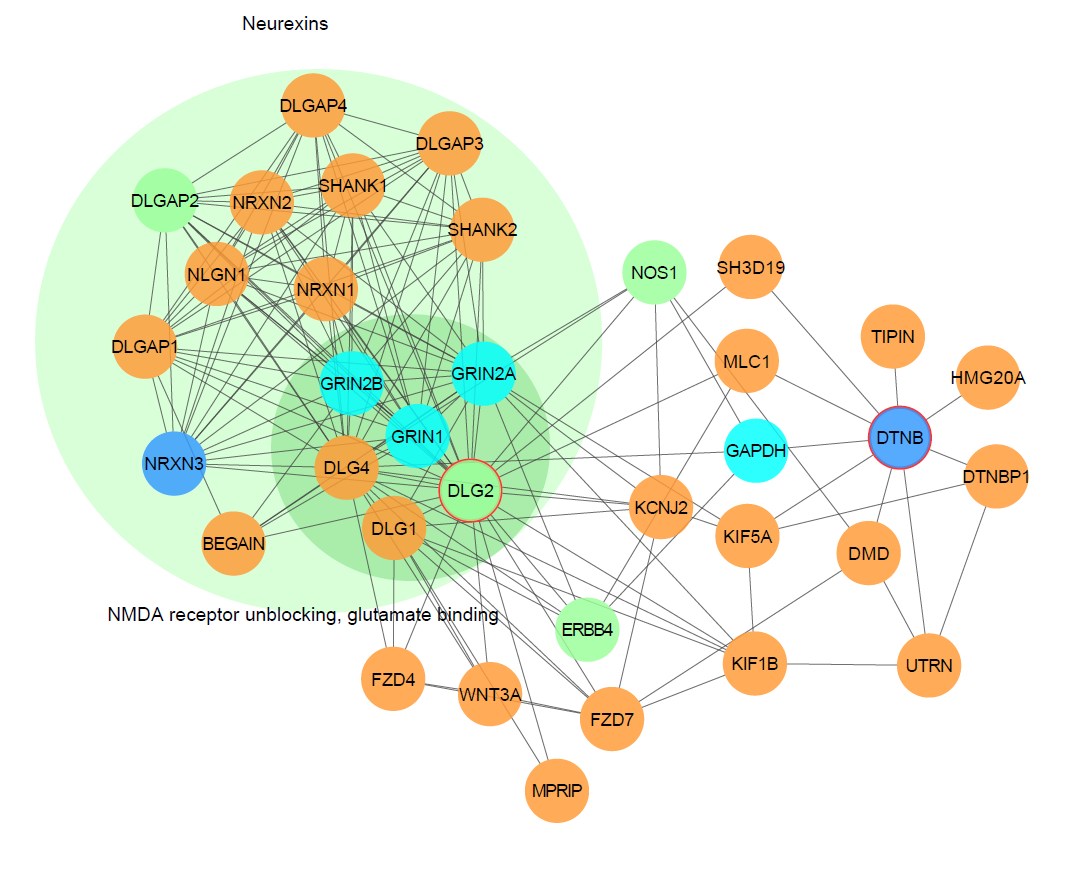


## Supplementary Tables

Supplementary Table 1: Subject characteristics.

| Cohort | Design | Type of sequencing | Num. families | N (n cases) | %female | | Mean (SD) age at onset in cases | Mean (SD) age at last exam in controls | APOE ε4 (rs429358) MAF %^1^ | |
| --- | --- | --- | --- | --- | --- | --- | --- | --- | --- | --- |
|  |  |  |  |  | Cases | Controls |  |  | Cases | Controls |
| NIMH | Family-based | WGS | 446 | 1393 (966) | 72.50% | 58.10% | 71.9(8.45) | 72.95(12.19) | 44.20% | 29.70% |
| NIA ADSP families | Family-based | WGS | 159 | 854 (543) | 61.90% | 63.90% | 72.61(8.53), 28 - age 90+ | 68.73(11.59), 8 - age 90+ | 22.40% | 16% |
| NIA ADSP unrelated NHW | Population-based | WGS | N/A | 1669 (983) | 44.90% | 57.70% | 74.81(8.82), 9 - age 90+ | 78.6(6.44), 18 - age 90+ | 33.10% | 12.80% |
| NIA ADSP unrelated AA | Population-based | WGS | N/A | 951 (450) | 68.40% | 74.10% | 76.7(7.80) | 80.9(6.10) | 34.60% | 16.50% |
| NIA ADSP unrelated HISP | Population-based | WGS | N/A | 1099 (486) | 68.10% | 70.60% | 76.7(7.06) | 73.0(7.45), 9 - age 90+ | 20.80% | 11.80% |

^1^APOE status was assessed based on WGS data. NIA ADSP families by design did not include individuals with two APOE-ε4 alleles.

Supplementary Table 2: Single variant test statistics in DTNB region.

| rsid | Discovery dataset (NIMH + NIA families) | | | Replication dataset NHW ADSP | | | Replication dataset AA ADSP | | | Replication dataset HISP ADSP | | |
| --- | --- | --- | --- | --- | --- | --- | --- | --- | --- | --- | --- | --- |
|  | EAF | Z-score | P-value | EAF | Z-score | P-value | EAF | Z-score | P-value | EAF | Z-score | P-value |
| rs375720932 | 0.001 | -2.397 | 1.65E-02 | 0.001 | -1.036 | 3.00E-01 | NA | NA | NA | NA | NA | NA |
| rs185521632 | 0.007 | -1.526 | 1.27E-01 | 0.008 | -1.752 | 7.98E-02 | NA | NA | NA | NA | NA | NA |
| rs183464131 | 0.004 | -1.698 | 8.96E-02 | NA | NA | NA | 0.013 | 0.123 | 9.02E-01 | 0.009 | -0.270 | 7.87E-01 |
| rs539056293 | NA | NA | NA | NA | NA | NA | 0.008 | 1.232 | 2.18E-01 | 0.002 | -0.524 | 6.00E-01 |
| rs115357042 | 0.004 | -1.698 | 8.96E-02 | NA | NA | NA | 0.016 | -0.169 | 8.66E-01 | 0.010 | 0.224 | 8.23E-01 |
| rs536783277 | NA | NA | NA | NA | NA | NA | 0.002 | 1.507 | 1.32E-01 | NA | NA | NA |
| rs150671624 | 0.004 | -1.698 | 8.96E-02 | NA | NA | NA | 0.015 | -0.116 | 9.07E-01 | 0.010 | 0.224 | 8.23E-01 |
| rs186440295 | 0.005 | -3.829 | 1.29E-04 | 0.002 | -1.479 | 1.39E-01 | NA | NA | NA | 0.004 | -0.841 | 4.00E-01 |
| rs577733912 | 0.001 | -1 | 3.17E-01 | 0.003 | 0.092 | 9.27E-01 | NA | NA | NA | NA | NA | NA |
| rs113414532 | NA | NA | NA | NA | NA | NA | 0.004 | 2.131 | 3.31E-02 | 0.002 | 0.524 | 6.00E-01 |

EAF - effect allele frequency. Variants with NAs were either excluded during QC or they had not enough samples (or informative families) to generate a single variant test statistic.

Supplementary Table 3: Single variant test statistics in DLG2 region.

| rsid | Discovery dataset (NIMH + NIA families) | | | Replication dataset NHW ADSP | | | Replication dataset AA ADSP | | | Replication dataset HISP ADSP | | |
| --- | --- | --- | --- | --- | --- | --- | --- | --- | --- | --- | --- | --- |
|  | EAF | Z-score | P-value | EAF | Z-score | P-value | EAF | Z-score | P-value | EAF | Z-score | P-value |
| rs141510844 | NA | NA | NA | NA | NA | NA | 0.023 | 1.704 | 8.84E-02 | 0.010 | -0.607 | 5.44E-01 |
| rs150332135 | NA | NA | NA | NA | NA | NA | 0.023 | 1.706 | 8.80E-02 | 0.010 | -0.607 | 5.44E-01 |
| rs182930441 | 0.004 | -0.707 | 4.80E-01 | NA | NA | NA | 0.007 | 0.998 | 3.18E-01 | 0.004 | -0.610 | 5.42E-01 |
| rs183182279 | 0.004 | -3.25 | 1.16E-03 | 0.004 | 1.396 | 1.63E-01 | NA | NA | NA | NA | NA | NA |
| rs187738292 | 0.005 | -2.898 | 3.76E-03 | 0.007 | 0.240 | 8.10E-01 | NA | NA | NA | NA | NA | NA |
| rs558771821 | NA | NA | NA | 0.002 | 1.725 | 8.45E-02 | NA | NA | NA | NA | NA | NA |
| rs541523762 | 0.002 | -1 | 3.17E-01 | NA | NA | NA | NA | NA | NA | 0.003 | 1.387 | 1.65E-01 |
| rs115037333 | NA | NA | NA | NA | NA | NA | 0.007 | -1.363 | 1.73E-01 | NA | NA | NA |
| rs147127256 | NA | NA | NA | NA | NA | NA | 0.023 | 1.702 | 8.87E-02 | 0.010 | -0.607 | 5.44E-01 |
| rs147410373 | 0.004 | 0.905 | 3.66E-01 | 0.005 | 1.255 | 2.10E-01 | 0.005 | 1.903 | 5.70E-02 | 0.003 | 0.164 | 8.69E-01 |

EAF - effect allele frequency. Variants with NAs were either excluded during QC or they had not enough samples (or informative families) to generate a single variant test statistic.

Supplementary Table 4: Differential expression for DLG2 and DTNB genes in the Alzheimer's Disease Dataset based on 253 human samples by Affymetrix Human Genome U133 Plus 2.0 Array.

| Gene symbol | Gene title | ID | t | B | logFC | P.Val | adj.P.Val |
| --- | --- | --- | --- | --- | --- | --- | --- |
| **All samples (80 AD cases and 173 controls, age > 20)** | | | | | | | |
| *DTNB* | dystrobrevin beta | 214253_s_at | -6.035 | 10.190 | -0.350 | 5.59E-09 | 1.35E-06 |
| *DLG2* | discs large MAGUK scaffold protein 2 | 228973_at | -4.421 | 2.764 | -0.228 | 1.46E-05 | 2.88E-04 |
| *DLG2* | discs large MAGUK scaffold protein 2 | 206253_at | -3.227 | -1.451 | -0.295 | 1.41E-03 | 8.57E-03 |
| *DTNB* | dystrobrevin beta | 215295_at | 1.166 | -5.782 | 0.071 | 2.45E-01 | 3.66E-01 |
| **All AD cases and controls with age > 50 (80 AD cases and 97 controls)** | | | | | | | |
| DTNB | dystrobrevin beta | 214253_s_at | -4.124 | 1.634 | -0.264 | 5.69E-05 | 9.10E-03 |
| DLG2 | discs large MAGUK scaffold protein 2 | 228973_at | -2.739 | -2.637 | -0.163 | 6.79E-03 | 6.96E-02 |
| DTNB | dystrobrevin beta | 215295_at | 1.751 | -4.707 | 0.129 | 8.16E-02 | 1.84E-01 |
| DLG2 | discs large MAGUK scaffold protein 2 | 206253_at | -0.591 | -6.002 | -0.059 | 5.55E-01 | 6.91E-01 |
| **All AD cases and controls with age > 80 (80 AD cases and 54 controls)** | | | | | | | |
| DTNB | dystrobrevin beta | 214253_s_at | -4.309 | 2.184 | -0.339 | 3.13E-05 | 2.45E-03 |
| DTNB | dystrobrevin beta | 215295_at | 3.713 | 0.128 | 0.304 | 2.98E-04 | 4.84E-03 |
| DLG2 | discs large MAGUK scaffold protein 2 | 228973_at | -2.900 | -2.270 | -0.209 | 4.35E-03 | 1.42E-02 |
| DLG2 | discs large MAGUK scaffold protein 2 | 206253_at | 0.034 | -6.215 | 0.004 | 9.73E-01 | 9.82E-01 |

Supplementary Table 5: Functional enrichments of genes interacting with DLG2 and DTNB.

| # background genes | # genes | category | description | FDR value | genes | term name |
| --- | --- | --- | --- | --- | --- | --- |
| 46 | 17 | NetworkNeighborAL | Neurexins and neuroligins, and Trafficking of AMPA receptors | 1.68E-32 | NRXN2, SHANK1, DLGAP1, DLG1, GRIN1, DLGAP3, DLGAP4, DLG2, GRIN2A, DLG4, NRXN1, NLGN1, DLGAP2, BEGAIN, NRXN3, SHANK2, GRIN2B | CL.8446 |
| 52 | 17 | NetworkNeighborAL | Neurexins and neuroligins, and Trafficking of AMPA receptors | 4.93E-32 | NRXN2, SHANK1, DLGAP1, DLG1, GRIN1, DLGAP3, DLGAP4, DLG2, GRIN2A, DLG4, NRXN1, NLGN1, DLGAP2, BEGAIN, NRXN3, SHANK2, GRIN2B | CL.8445 |
| 55 | 16 | Reactome Pathways | Neurexins and neuroligins | 6.03E-29 | NRXN2, SHANK1, DLGAP1, GRIN1, DLGAP3, DLGAP4, DLG2, GRIN2A, DLG4, NRXN1, NLGN1, DLGAP2, BEGAIN, NRXN3, SHANK2, GRIN2B | HSA-6794361 |
| 85 | 17 | Reactome Pathways | Protein-protein interactions at synapses | 8.56E-29 | NRXN2, SHANK1, DLGAP1, DLG1, GRIN1, DLGAP3, DLGAP4, DLG2, GRIN2A, DLG4, NRXN1, NLGN1, DLGAP2, BEGAIN, NRXN3, SHANK2, GRIN2B | HSA-6794362 |
| 153 | 17 | NetworkNeighborAL | mixed, incl. Postsynaptic cell membrane, and Chloride channel | 3.08E-25 | NRXN2, SHANK1, DLGAP1, DLG1, GRIN1, DLGAP3, DLGAP4, DLG2, GRIN2A, DLG4, NRXN1, NLGN1, DLGAP2, BEGAIN, NRXN3, SHANK2, GRIN2B | CL.8440 |
| 18 | 11 | NetworkNeighborAL | Neurexins and neuroligins | 5.69E-23 | NRXN2, SHANK1, DLGAP1, DLGAP3, DLGAP4, DLG4, NRXN1, NLGN1, DLGAP2, NRXN3, SHANK2 | CL.8499 |
| 358 | 18 | Reactome Pathways | Neuronal System | 1.02E-20 | KCNJ2, NRXN2, SHANK1, DLGAP1, DLG1, GRIN1, DLGAP3, DLGAP4, DLG2, GRIN2A, DLG4, NRXN1, NLGN1, DLGAP2, BEGAIN, NRXN3, SHANK2, GRIN2B | HSA-112316 |
| 849 | 22 | GO Component | synapse | 3.26E-19 | KCNJ2, NRXN2, WNT3A, SHANK1, DLGAP1, DTNBP1, DLG1, DMD, UTRN, GRIN1, DLGAP3, DLGAP4, DLG2, GRIN2A, DLG4, DTNB, NRXN1, NLGN1, DLGAP2, SHANK2, GRIN2B, NOS1 | GO.0045202 |
| 199 | 15 | UniProt Keywords | Postsynaptic cell membrane | 6.88E-19 | SHANK1, DLGAP1, DTNBP1, DLG1, DMD, UTRN, GRIN1, DLGAP3, DLG2, GRIN2A, DLG4, NLGN1, DLGAP2, SHANK2, GRIN2B | KW-0628 |
| 705 | 20 | GO Component | synapse part | 4.76E-18 | KCNJ2, NRXN2, WNT3A, SHANK1, DLGAP1, DTNBP1, DLG1, DMD, UTRN, GRIN1, DLGAP3, DLG2, GRIN2A, DLG4, NRXN1, NLGN1, DLGAP2, SHANK2, GRIN2B, NOS1 | GO.0044456 |
| 237 | 15 | GO Component | postsynaptic membrane | 6.08E-18 | SHANK1, DLGAP1, DTNBP1, DLG1, DMD, UTRN, GRIN1, DLGAP3, DLG2, GRIN2A, DLG4, NLGN1, DLGAP2, SHANK2, GRIN2B | GO.0045211 |
| 308 | 16 | GO Component | synaptic membrane | 6.08E-18 | SHANK1, DLGAP1, DTNBP1, DLG1, DMD, UTRN, GRIN1, DLGAP3, DLG2, GRIN2A, DLG4, NRXN1, NLGN1, DLGAP2, SHANK2, GRIN2B | GO.0097060 |
| 435 | 17 | GO Component | postsynapse | 1.89E-17 | KCNJ2, SHANK1, DLGAP1, DTNBP1, DLG1, DMD, UTRN, GRIN1, DLGAP3, DLG2, GRIN2A, DLG4, NLGN1, DLGAP2, SHANK2, GRIN2B, NOS1 | GO.0098794 |
| 1449 | 23 | GO Component | neuron part | 1.88E-16 | KCNJ2, KIF1B, NRXN2, WNT3A, SHANK1, DLGAP1, DTNBP1, DLG1, DMD, UTRN, GRIN1, DLGAP3, DLG2, GRIN2A, DLG4, NRXN1, NLGN1, DLGAP2, KIF5A, FZD4, SHANK2, GRIN2B, NOS1 | GO.0097458 |
| 399 | 16 | UniProt Keywords | Synapse | 1.94E-16 | SHANK1, DLGAP1, DTNBP1, DLG1, DMD, UTRN, GRIN1, DLGAP3, DLG2, GRIN2A, DLG4, NRXN1, NLGN1, DLGAP2, SHANK2, GRIN2B | KW-0770 |
| 205 | 13 | GO Component | postsynaptic density | 1.31E-15 | SHANK1, DLGAP1, DTNBP1, DLG1, GRIN1, DLGAP3, DLG2, GRIN2A, DLG4, NLGN1, DLGAP2, SHANK2, GRIN2B | GO.0014069 |
| 40 | 10 | GO Process | protein localization to synapse | 1.46E-15 | NRXN2, SHANK1, DLG1, GRIN1, DLG2, GRIN2A, DLG4, NRXN1, NLGN1, KIF5A | GO.0035418 |
| 1006 | 19 | GO Component | cell junction | 1.76E-14 | KCNJ2, SHANK1, MLC1, DLGAP1, DTNBP1, DLG1, DMD, UTRN, GRIN1, DLGAP3, DLG2, GRIN2A, DLG4, NRXN1, NLGN1, DLGAP2, FZD4, SHANK2, GRIN2B | GO.0030054 |
| 12 | 7 | NetworkNeighborAL | mixed, incl. SAPAP family, and GKAP/Homer scaffold activity | 2.14E-14 | SHANK1, DLGAP1, DLGAP3, DLGAP4, DLG4, DLGAP2, SHANK2 | CL.8500 |
| 1061 | 19 | GO Component | plasma membrane region | 4.20E-14 | SHANK1, MLC1, DLGAP1, DTNBP1, ERBB4, DLG1, DMD, UTRN, GRIN1, DLGAP3, DLG2, GRIN2A, DLG4, NRXN1, NLGN1, DLGAP2, SHANK2, GRIN2B, NOS1 | GO.0098590 |
| 1142 | 18 | GO Component | neuron projection | 2.59E-12 | KCNJ2, KIF1B, SHANK1, DTNBP1, DLG1, DMD, UTRN, GRIN1, DLG2, GRIN2A, DLG4, NRXN1, NLGN1, KIF5A, FZD4, SHANK2, GRIN2B, NOS1 | GO.0043005 |
| 408 | 14 | GO Process | trans-synaptic signaling | 5.34E-12 | KIF1B, NRXN2, DLGAP1, DLG1, GRIN1, DLG2, GRIN2A, DLG4, NRXN1, NLGN1, DLGAP2, KIF5A, GRIN2B, NOS1 | GO.0099537 |
| 18 | 6 | NetworkNeighborAL | Unblocking of NMDA receptors, glutamate binding and activation, and eye blink reflex | 3.36E-11 | DLG1, GRIN1, DLG2, GRIN2A, BEGAIN, GRIN2B | CL.8449 |
| 2651 | 23 | GO Component | plasma membrane part | 3.65E-11 | KCNJ2, WNT3A, SHANK1, MLC1, DLGAP1, DTNBP1, ERBB4, DLG1, DMD, UTRN, GRIN1, DLGAP3, DLG2, GRIN2A, DLG4, NRXN1, NLGN1, DLGAP2, FZD4, NRXN3, SHANK2, GRIN2B, NOS1 | GO.0044459 |
| 402 | 13 | GO Process | chemical synaptic transmission | 6.46E-11 | KIF1B, NRXN2, DLGAP1, DLG1, GRIN1, DLG2, GRIN2A, DLG4, NRXN1, NLGN1, DLGAP2, KIF5A, GRIN2B | GO.0007268 |
| 18 | 6 | Reactome Pathways | CREB phosphorylation through the activation of CaMKII | 7.46E-11 | DLG1, GRIN1, DLG2, GRIN2A, DLG4, GRIN2B | HSA-442729 |
| 20 | 6 | Reactome Pathways | Unblocking of NMDA receptors, glutamate binding and activation | 1.02E-10 | DLG1, GRIN1, DLG2, GRIN2A, DLG4, GRIN2B | HSA-438066 |
| 20 | 6 | Reactome Pathways | Ras activation upon Ca2+ influx through NMDA receptor | 1.02E-10 | DLG1, GRIN1, DLG2, GRIN2A, DLG4, GRIN2B | HSA-442982 |
| 1073 | 17 | GO Process | cell-cell signaling | 2.25E-10 | KIF1B, NRXN2, WNT3A, FZD7, DLGAP1, DLG1, GRIN1, DLG2, GRIN2A, DLG4, NRXN1, NLGN1, DLGAP2, KIF5A, FZD4, GRIN2B, NOS1 | GO.0007267 |
| 10 | 5 | NetworkNeighborAL | glutamate-gated calcium ion channel activity, and Disks large 1-like | 3.95E-10 | GRIN1, DLG2, GRIN2A, BEGAIN, GRIN2B | CL.8450 |
| 16 | 6 | GO Process | vocalization behavior | 4.03E-10 | NRXN2, SHANK1, DLG4, NRXN1, NRXN3, SHANK2 | GO.0071625 |
| 30 | 6 | Reactome Pathways | CREB phosphorylation through the activation of Ras | 6.08E-10 | DLG1, GRIN1, DLG2, GRIN2A, DLG4, GRIN2B | HSA-442742 |
| 10 | 5 | GO Component | NMDA selective glutamate receptor complex | 7.40E-10 | SHANK1, GRIN1, GRIN2A, NLGN1, GRIN2B | GO.0017146 |
| 5159 | 28 | GO Component | plasma membrane | 9.69E-10 | GAPDH, KCNJ2, NRXN2, WNT3A, FZD7, SHANK1, SH3D19, MLC1, DLGAP1, DTNBP1, ERBB4, DLG1, DMD, UTRN, GRIN1, DLGAP3, DLGAP4, DLG2, GRIN2A, DLG4, NRXN1, NLGN1, DLGAP2, FZD4, NRXN3, SHANK2, GRIN2B, NOS1 | GO.0005886 |
| 2206 | 21 | GO Process | nervous system development | 1.49E-09 | NRXN2, WNT3A, FZD7, SHANK1, DTNBP1, ERBB4, DLG1, DMD, GRIN1, DLG2, HMG20A, GRIN2A, DLG4, NRXN1, NLGN1, KIF5A, FZD4, NRXN3, SHANK2, GRIN2B, NOS1 | GO.0007399 |
| 50 | 7 | GO Process | social behavior | 1.58E-09 | NRXN2, SHANK1, GRIN1, DLG4, NRXN1, NRXN3, SHANK2 | GO.0035176 |
| 23 | 6 | GO Process | receptor localization to synapse | 1.62E-09 | DLG1, DLG2, DLG4, NRXN1, NLGN1, KIF5A | GO.0097120 |
| 39 | 6 | Reactome Pathways | Post NMDA receptor activation events | 2.20E-09 | DLG1, GRIN1, DLG2, GRIN2A, DLG4, GRIN2B | HSA-438064 |
| 41 | 6 | GO Component | ionotropic glutamate receptor complex | 3.14E-09 | SHANK1, GRIN1, GRIN2A, DLG4, NLGN1, GRIN2B | GO.0008328 |
| 143 | 8 | GO Component | dendritic spine | 3.14E-09 | KCNJ2, SHANK1, DTNBP1, GRIN1, DLG4, NLGN1, SHANK2, NOS1 | GO.0043197 |
| 1316 | 16 | GO Component | cell projection part | 3.14E-09 | KCNJ2, KIF1B, SHANK1, DTNBP1, DLG1, DMD, UTRN, GRIN1, DLG2, DLG4, NRXN1, NLGN1, KIF5A, FZD4, SHANK2, NOS1 | GO.0044463 |
| 1316 | 16 | GO Component | plasma membrane bounded cell projection part | 3.14E-09 | KCNJ2, KIF1B, SHANK1, DTNBP1, DLG1, DMD, UTRN, GRIN1, DLG2, DLG4, NRXN1, NLGN1, KIF5A, FZD4, SHANK2, NOS1 | GO.0120038 |
| 43 | 6 | Reactome Pathways | Activation of NMDA receptors and postsynaptic events | 3.34E-09 | DLG1, GRIN1, DLG2, GRIN2A, DLG4, GRIN2B | HSA-442755 |
| 341 | 10 | GO Component | axon part | 4.26E-09 | KIF1B, DTNBP1, DLG1, UTRN, GRIN1, DLG2, DLG4, NRXN1, KIF5A, SHANK2 | GO.0033267 |
| 66 | 7 | GO Process | multi-organism behavior | 7.14E-09 | NRXN2, SHANK1, GRIN1, DLG4, NRXN1, NRXN3, SHANK2 | GO.0051705 |
| 502 | 11 | GO Component | plasma membrane protein complex | 8.97E-09 | KCNJ2, WNT3A, SHANK1, DLG1, DMD, UTRN, GRIN1, GRIN2A, DLG4, NLGN1, GRIN2B | GO.0098797 |
| 21 | 5 | Reactome Pathways | Synaptic adhesion-like molecules | 9.49E-09 | DLG1, GRIN1, GRIN2A, DLG4, GRIN2B | HSA-8849932 |
| 5 | 4 | NetworkNeighborAL | glutamate-gated calcium ion channel activity, and Disks large 1-like | 9.60E-09 | GRIN1, DLG2, GRIN2A, GRIN2B | CL.8455 |
| 126 | 8 | GO Process | positive regulation of synaptic transmission | 1.12E-08 | SHANK1, DTNBP1, GRIN1, GRIN2A, DLG4, NRXN1, NLGN1, SHANK2 | GO.0050806 |
| 6 | 4 | NetworkNeighborAL | postsynaptic membrane assembly | 1.46E-08 | NRXN2, NRXN1, NLGN1, NRXN3 | CL.8523 |
| 137 | 8 | GO Process | learning | 1.96E-08 | NRXN2, SHANK1, GRIN1, GRIN2A, DLG4, NRXN1, NRXN3, SHANK2 | GO.0007612 |
| 40 | 6 | GO Process | receptor clustering | 2.29E-08 | NRXN2, DLG1, DLG2, DLG4, NRXN1, NLGN1 | GO.0043113 |
| 706 | 13 | GO Function | protein domain specific binding | 3.23E-08 | GAPDH, WNT3A, FZD7, SHANK1, DLGAP1, DLG1, DLGAP3, DLGAP4, DLG4, NLGN1, DLGAP2, FZD4, SHANK2 | GO.0019904 |
| 6605 | 30 | GO Function | protein binding | 3.92E-08 | GAPDH, KCNJ2, KIF1B, NRXN2, WNT3A, FZD7, SHANK1, SH3D19, DLGAP1, ERBB4, DLG1, DMD, UTRN, GRIN1, DLGAP3, DLGAP4, DLG2, HMG20A, MPRIP, GRIN2A, DLG4, NRXN1, NLGN1, DLGAP2, KIF5A, FZD4, NRXN3, SHANK2, GRIN2B, NOS1 | GO.0005515 |
| 237 | 9 | GO Process | learning or memory | 4.15E-08 | NRXN2, SHANK1, GRIN1, GRIN2A, DLG4, NRXN1, NRXN3, SHANK2, GRIN2B | GO.0007611 |
| 94 | 7 | GO Process | localization within membrane | 5.14E-08 | NRXN2, DLG1, DLG2, DLG4, NRXN1, NLGN1, KIF5A | GO.0051668 |
| 5 | 4 | Pfam | Guanylate-kinase-associated protein (GKAP) protein | 6.50E-08 | DLGAP1, DLGAP3, DLGAP4, DLGAP2 | PF03359 |
| 5 | 4 | GO Function | neuroligin family protein binding | 8.39E-08 | NRXN2, DLG4, NRXN1, NRXN3 | GO.0097109 |
| 112 | 7 | KEGG Pathways | Glutamatergic synapse | 8.92E-08 | SHANK1, DLGAP1, GRIN1, GRIN2A, DLG4, SHANK2, GRIN2B | hsa04724 |
| 150 | 7 | Reactome Pathways | Neurotransmitter receptors and postsynaptic signal transmission | 1.03E-07 | KCNJ2, DLG1, GRIN1, DLG2, GRIN2A, DLG4, GRIN2B | HSA-112314 |
| 5 | 4 | InterPro Domains | SAPAP family | 1.15E-07 | DLGAP1, DLGAP3, DLGAP4, DLGAP2 | IPR005026 |
| 516 | 11 | GO Process | regulation of system process | 1.16E-07 | KCNJ2, SHANK1, DLG1, DMD, UTRN, GRIN1, GRIN2A, DLG4, NRXN1, NLGN1, NOS1 | GO.0044057 |
| 189 | 8 | GO Process | synapse organization | 1.58E-07 | NRXN2, WNT3A, SHANK1, UTRN, DLG4, NRXN1, NLGN1, SHANK2 | GO.0050808 |
| 1519 | 16 | GO Process | neurogenesis | 1.60E-07 | WNT3A, FZD7, SHANK1, DTNBP1, ERBB4, DMD, GRIN1, HMG20A, GRIN2A, DLG4, NRXN1, NLGN1, KIF5A, FZD4, NRXN3, NOS1 | GO.0022008 |
| 408 | 10 | GO Process | regulation of membrane potential | 1.70E-07 | KCNJ2, SHANK1, DLG1, DMD, GRIN1, GRIN2A, DLG4, NRXN1, NLGN1, GRIN2B | GO.0042391 |
| 294 | 9 | GO Process | regulation of cation transmembrane transport | 1.80E-07 | KCNJ2, SHANK1, DLG1, DMD, UTRN, DLG4, NRXN1, NLGN1, NOS1 | GO.1904062 |
| 26 | 5 | GO Process | positive regulation of excitatory postsynaptic potential | 1.80E-07 | SHANK1, GRIN1, DLG4, NRXN1, NLGN1 | GO.2000463 |
| 28 | 5 | GO Function | ionotropic glutamate receptor binding | 1.82E-07 | SHANK1, DLG1, DLG2, DLG4, SHANK2 | GO.0035255 |
| 3208 | 21 | UniProt Keywords | Cell membrane | 2.06E-07 | FZD7, SHANK1, MLC1, DLGAP1, DTNBP1, ERBB4, DLG1, DMD, UTRN, GRIN1, DLGAP3, DLG2, GRIN2A, DLG4, NRXN1, NLGN1, DLGAP2, FZD4, SHANK2, GRIN2B, NOS1 | KW-1003 |
| 1827 | 17 | GO Process | system process | 2.15E-07 | KCNJ2, NRXN2, SHANK1, DLG1, DMD, UTRN, GRIN1, DLG2, GRIN2A, DLG4, NRXN1, NLGN1, FZD4, NRXN3, SHANK2, GRIN2B, NOS1 | GO.0003008 |
| 530 | 10 | GO Component | axon | 2.27E-07 | KIF1B, DTNBP1, DLG1, UTRN, GRIN1, DLG2, DLG4, NRXN1, KIF5A, SHANK2 | GO.0030424 |
| 531 | 10 | GO Component | dendrite | 2.27E-07 | KCNJ2, SHANK1, DTNBP1, GRIN1, DLG4, NLGN1, KIF5A, FZD4, SHANK2, NOS1 | GO.0030425 |
| 316 | 9 | GO Process | modulation of chemical synaptic transmission | 2.95E-07 | SHANK1, DTNBP1, GRIN1, GRIN2A, DLG4, NRXN1, NLGN1, SHANK2, GRIN2B | GO.0050804 |
| 731 | 11 | GO Component | somatodendritic compartment | 3.46E-07 | KCNJ2, SHANK1, DTNBP1, GRIN1, DLG4, NRXN1, NLGN1, KIF5A, FZD4, SHANK2, NOS1 | GO.0036477 |
| 224 | 8 | GO Process | regulation of ion transmembrane transporter activity | 4.08E-07 | SHANK1, DLG1, DMD, UTRN, DLG4, NRXN1, NLGN1, NOS1 | GO.0032412 |
| 618 | 11 | GO Process | regulation of ion transport | 4.55E-07 | KCNJ2, SHANK1, DTNBP1, DLG1, DMD, UTRN, GRIN1, DLG4, NRXN1, NLGN1, NOS1 | GO.0043269 |
| 1422 | 15 | GO Process | generation of neurons | 4.55E-07 | WNT3A, FZD7, SHANK1, DTNBP1, ERBB4, DMD, GRIN1, HMG20A, DLG4, NRXN1, NLGN1, KIF5A, FZD4, NRXN3, NOS1 | GO.0048699 |
| 817 | 12 | GO Process | regulation of nervous system development | 6.02E-07 | WNT3A, SHANK1, ERBB4, DLG1, DMD, GRIN1, HMG20A, DLG4, NRXN1, NLGN1, FZD4, NOS1 | GO.0051960 |
| 152 | 7 | GO Process | regulation of cation channel activity | 6.20E-07 | SHANK1, DLG1, DMD, DLG4, NRXN1, NLGN1, NOS1 | GO.2001257 |
| 206 | 7 | GO Component | cation channel complex | 7.32E-07 | KCNJ2, SHANK1, GRIN1, GRIN2A, DLG4, NLGN1, GRIN2B | GO.0034703 |
| 122 | 6 | GO Component | sarcolemma | 8.07E-07 | KCNJ2, DTNBP1, DLG1, DMD, UTRN, NOS1 | GO.0042383 |
| 218 | 7 | Reactome Pathways | Transmission across Chemical Synapses | 1.12E-06 | KCNJ2, DLG1, GRIN1, DLG2, GRIN2A, DLG4, GRIN2B | HSA-112315 |
| 14 | 4 | GO Process | neuron cell-cell adhesion | 1.33E-06 | NRXN2, NRXN1, NLGN1, NRXN3 | GO.0007158 |
| 541 | 10 | GO Process | behavior | 1.40E-06 | NRXN2, SHANK1, GRIN1, GRIN2A, DLG4, NRXN1, FZD4, NRXN3, SHANK2, GRIN2B | GO.0007610 |
| 354 | 8 | GO Component | presynapse | 1.56E-06 | NRXN2, WNT3A, DTNBP1, GRIN1, GRIN2A, DLG4, NRXN1, NLGN1 | GO.0098793 |
| 8420 | 30 | GO Component | membrane | 1.81E-06 | GAPDH, KCNJ2, KIF1B, NRXN2, WNT3A, FZD7, SHANK1, SH3D19, MLC1, DLGAP1, DTNBP1, ERBB4, DLG1, DMD, UTRN, GRIN1, DLGAP3, DLGAP4, DLG2, GRIN2A, DLG4, NRXN1, NLGN1, DLGAP2, BEGAIN, FZD4, NRXN3, SHANK2, GRIN2B, NOS1 | GO.0016020 |
| 71 | 5 | GO Component | neuron projection cytoplasm | 1.81E-06 | KIF1B, DTNBP1, DLG2, DLG4, KIF5A | GO.0120111 |
| 5 | 3 | NetworkNeighborAL | phospholipase C-activating G protein-coupled glutamate receptor signaling pathway, and Disks large-associated protein 1 | 2.21E-06 | SHANK1, DLGAP1, DLG4 | CL.8502 |
| 29 | 4 | GO Component | excitatory synapse | 2.52E-06 | SHANK1, GRIN1, DLG4, NLGN1 | GO.0060076 |
| 112 | 6 | GO Process | regulation of neurological system process | 2.90E-06 | SHANK1, GRIN1, GRIN2A, DLG4, NRXN1, NLGN1 | GO.0031644 |
| 54 | 5 | GO Process | postsynapse organization | 2.95E-06 | NRXN2, SHANK1, DLG4, NRXN1, NLGN1 | GO.0099173 |
| 131 | 6 | Pfam | PDZ domain (Also known as DHR or GLGF) | 3.17E-06 | SHANK1, DLG1, DLG2, DLG4, SHANK2, NOS1 | PF00595 |
| 4 | 3 | Pfam | PDZ-associated domain of NMDA receptors | 3.17E-06 | DLG1, DLG2, DLG4 | PF10600 |
| 3 | 3 | Pfam | Polyubiquitination (PEST) N-terminal domain of MAGUK | 3.17E-06 | DLG1, DLG2, DLG4 | PF10608 |
| 57 | 5 | GO Function | scaffold protein binding | 3.19E-06 | KIF1B, SHANK1, DLG4, NLGN1, NOS1 | GO.0097110 |
| 1513 | 14 | GO Function | signaling receptor binding | 4.69E-06 | NRXN2, WNT3A, FZD7, SHANK1, ERBB4, DLG1, UTRN, GRIN1, DLG2, DLG4, NRXN1, NLGN1, NRXN3, SHANK2 | GO.0005102 |
| 148 | 6 | SMART Domains | Domain present in PSD-95, Dlg, and ZO-1/2. | 4.72E-06 | SHANK1, DLG1, DLG2, DLG4, SHANK2, NOS1 | SM00228 |
| 4 | 3 | SMART Domains | Polyubiquitination (PEST) N-terminal domain of MAGUK | 4.72E-06 | DLG1, DLG2, DLG4 | SM01277 |
| 626 | 10 | GO Process | cell morphogenesis | 4.74E-06 | WNT3A, FZD7, SHANK1, DTNBP1, DLG4, NRXN1, NLGN1, KIF5A, FZD4, NRXN3 | GO.0000902 |
| 4144 | 22 | GO Process | system development | 5.24E-06 | NRXN2, WNT3A, FZD7, SHANK1, DTNBP1, ERBB4, DLG1, DMD, UTRN, GRIN1, DLG2, HMG20A, GRIN2A, DLG4, NRXN1, NLGN1, KIF5A, FZD4, NRXN3, SHANK2, GRIN2B, NOS1 | GO.0048731 |
| 167 | 6 | Pfam | SH3 domain | 5.31E-06 | SHANK1, SH3D19, DLG1, DLG2, DLG4, SHANK2 | PF00018 |
| 7 | 3 | Pfam | Syndecan domain | 5.31E-06 | NRXN2, NRXN1, NRXN3 | PF01034 |
| 171 | 6 | Pfam | Variant SH3 domain | 5.31E-06 | SHANK1, SH3D19, DLG1, DLG2, DLG4, SHANK2 | PF07653 |
| 6 | 3 | Pfam | EF hand | 5.31E-06 | DMD, UTRN, DTNB | PF09068 |
| 6 | 3 | Pfam | EF-hand | 5.31E-06 | DMD, UTRN, DTNB | PF09069 |
| 3 | 3 | InterPro Domains | Neurexin | 5.61E-06 | NRXN2, NRXN1, NRXN3 | IPR037440 |
| 140 | 6 | GO Function | ligand-gated ion channel activity | 6.17E-06 | KCNJ2, DLG1, GRIN1, GRIN2A, DLG4, GRIN2B | GO.0015276 |
| 150 | 6 | InterPro Domains | PDZ domain | 6.54E-06 | SHANK1, DLG1, DLG2, DLG4, SHANK2, NOS1 | IPR001478 |
| 6 | 3 | InterPro Domains | EF-hand domain, type 1 | 6.54E-06 | DMD, UTRN, DTNB | IPR015153 |
| 6 | 3 | InterPro Domains | EF-hand domain, type 2 | 6.54E-06 | DMD, UTRN, DTNB | IPR015154 |
| 4 | 3 | InterPro Domains | Disks large 1-like | 6.54E-06 | DLG1, DLG2, DLG4 | IPR016313 |
| 4 | 3 | InterPro Domains | PDZ-associated domain of NMDA receptors | 6.54E-06 | DLG1, DLG2, DLG4 | IPR019583 |
| 4 | 3 | InterPro Domains | Disks large homologue 1, N-terminal PEST domain | 6.54E-06 | DLG1, DLG2, DLG4 | IPR019590 |
| 151 | 6 | InterPro Domains | PDZ superfamily | 6.54E-06 | SHANK1, DLG1, DLG2, DLG4, SHANK2, NOS1 | IPR036034 |
| 7 | 3 | InterPro Domains | Syndecan/Neurexin domain | 6.70E-06 | NRXN2, NRXN1, NRXN3 | IPR027789 |
| 5 | 3 | GO Function | glutamate-gated calcium ion channel activity | 6.85E-06 | GRIN1, GRIN2A, GRIN2B | GO.0022849 |
| 24 | 4 | GO Process | postsynaptic membrane organization | 7.20E-06 | NRXN2, DLG4, NRXN1, NLGN1 | GO.0001941 |
| 305 | 7 | GO Component | receptor complex | 7.53E-06 | SHANK1, ERBB4, GRIN1, GRIN2A, DLG4, NLGN1, GRIN2B | GO.0043235 |
| 498 | 9 | GO Process | cell morphogenesis involved in differentiation | 7.70E-06 | WNT3A, FZD7, SHANK1, DTNBP1, DLG4, NRXN1, KIF5A, FZD4, NRXN3 | GO.0000904 |
| 137 | 6 | GO Process | adult behavior | 7.70E-06 | NRXN2, SHANK1, GRIN1, NRXN1, NRXN3, SHANK2 | GO.0030534 |
| 10 | 3 | GO Component | postsynaptic density membrane | 9.31E-06 | GRIN1, GRIN2A, DLG4 | GO.0098839 |
| 152 | 6 | KEGG Pathways | Hippo signaling pathway | 1.00E-05 | WNT3A, FZD7, DLG1, DLG2, DLG4, FZD4 | hsa04390 |
| 11 | 3 | GO Component | integral component of synaptic membrane | 1.14E-05 | GRIN2A, NRXN1, NLGN1 | GO.0099699 |
| 46 | 4 | GO Component | axon cytoplasm | 1.14E-05 | KIF1B, DTNBP1, DLG2, KIF5A | GO.1904115 |
| 77 | 5 | GO Process | synapse assembly | 1.30E-05 | NRXN2, WNT3A, NRXN1, NLGN1, SHANK2 | GO.0007416 |
| 1367 | 13 | GO Process | cellular protein localization | 1.30E-05 | KIF1B, NRXN2, WNT3A, SHANK1, DLG1, DMD, GRIN1, DLG2, GRIN2A, DLG4, NRXN1, NLGN1, KIF5A | GO.0034613 |
| 7 | 3 | GO Function | NMDA glutamate receptor activity | 1.33E-05 | GRIN1, GRIN2A, GRIN2B | GO.0004972 |
| 720 | 10 | GO Process | cellular component morphogenesis | 1.40E-05 | WNT3A, FZD7, SHANK1, DTNBP1, DLG4, NRXN1, NLGN1, KIF5A, FZD4, NRXN3 | GO.0032989 |
| 730 | 10 | GO Process | regulation of neurogenesis | 1.55E-05 | WNT3A, SHANK1, ERBB4, DMD, GRIN1, HMG20A, DLG4, NLGN1, FZD4, NOS1 | GO.0050767 |
| 6 | 3 | GO Process | postsynaptic density protein 95 clustering | 1.55E-05 | NRXN2, NRXN1, NLGN1 | GO.0097119 |
| 6984 | 27 | UniProt Keywords | Membrane | 1.57E-05 | GAPDH, KCNJ2, NRXN2, FZD7, SHANK1, MLC1, DLGAP1, DTNBP1, ERBB4, DLG1, DMD, UTRN, GRIN1, DLGAP3, DLGAP4, DLG2, GRIN2A, DLG4, NRXN1, NLGN1, DLGAP2, BEGAIN, FZD4, NRXN3, SHANK2, GRIN2B, NOS1 | KW-0472 |
| 940 | 11 | GO Process | neuron differentiation | 1.64E-05 | WNT3A, FZD7, SHANK1, DTNBP1, ERBB4, DLG4, NRXN1, NLGN1, KIF5A, FZD4, NRXN3 | GO.0030182 |
| 164 | 6 | GO Process | regulation of synaptic plasticity | 1.75E-05 | GRIN1, GRIN2A, DLG4, NLGN1, SHANK2, GRIN2B | GO.0048167 |
| 405 | 8 | GO Process | protein localization to membrane | 1.75E-05 | NRXN2, DLG1, GRIN1, DLG2, GRIN2A, DLG4, NRXN1, NLGN1 | GO.0072657 |
| 7 | 3 | GO Process | neurotransmitter-gated ion channel clustering | 2.04E-05 | DLG4, NRXN1, NLGN1 | GO.0072578 |
| 215 | 6 | SMART Domains | Src homology 3 domains | 2.05E-05 | SHANK1, SH3D19, DLG1, DLG2, DLG4, SHANK2 | SM00326 |
| 212 | 6 | InterPro Domains | SH3-like domain superfamily | 2.15E-05 | SHANK1, SH3D19, DLG1, DLG2, DLG4, SHANK2 | IPR036028 |
| 766 | 10 | GO Process | regulation of cellular localization | 2.15E-05 | WNT3A, MLC1, DTNBP1, ERBB4, DLG1, DMD, GRIN1, NRXN1, NLGN1, NOS1 | GO.0060341 |
| 14 | 3 | SMART Domains | putative band 4.1 homologues' binding motif | 2.27E-05 | NRXN2, NRXN1, NRXN3 | SM00294 |
| 1732 | 14 | GO Process | regulation of transport | 2.29E-05 | GAPDH, KCNJ2, WNT3A, SHANK1, MLC1, DTNBP1, DLG1, DMD, UTRN, GRIN1, DLG4, NRXN1, NLGN1, NOS1 | GO.0051049 |
| 222 | 6 | InterPro Domains | SH3 domain | 2.56E-05 | SHANK1, SH3D19, DLG1, DLG2, DLG4, SHANK2 | IPR001452 |
| 6517 | 25 | GO Component | membrane part | 2.64E-05 | KCNJ2, NRXN2, WNT3A, FZD7, SHANK1, MLC1, DLGAP1, DTNBP1, ERBB4, DLG1, DMD, UTRN, GRIN1, DLGAP3, DLG2, GRIN2A, DLG4, NRXN1, NLGN1, DLGAP2, FZD4, NRXN3, SHANK2, GRIN2B, NOS1 | GO.0044425 |
| 8 | 3 | GO Process | excitatory chemical synaptic transmission | 2.66E-05 | GRIN1, GRIN2A, GRIN2B | GO.0098976 |
| 600 | 9 | GO Process | regulation of plasma membrane bounded cell projection organization | 2.66E-05 | WNT3A, SHANK1, DMD, GRIN1, DLG4, NRXN1, NLGN1, FZD4, GRIN2B | GO.0120035 |
| 229 | 6 | Reactome Pathways | RAF/MAP kinase cascade | 2.86E-05 | DLG1, GRIN1, DLG2, GRIN2A, DLG4, GRIN2B | HSA-5673001 |
| 14 | 3 | InterPro Domains | Neurexin/syndecan/glycophorin C | 2.90E-05 | NRXN2, NRXN1, NRXN3 | IPR003585 |
| 234 | 6 | Reactome Pathways | MAPK1/MAPK3 signaling | 3.00E-05 | DLG1, GRIN1, DLG2, GRIN2A, DLG4, GRIN2B | HSA-5684996 |
| 5108 | 23 | GO Process | signaling | 3.04E-05 | KCNJ2, KIF1B, NRXN2, WNT3A, FZD7, DLGAP1, ERBB4, DLG1, DMD, GRIN1, DLGAP3, DLGAP4, DLG2, GRIN2A, DLG4, NRXN1, NLGN1, DLGAP2, KIF5A, FZD4, NRXN3, GRIN2B, NOS1 | GO.0023052 |
| 448 | 8 | GO Process | neuron projection morphogenesis | 3.10E-05 | WNT3A, SHANK1, DTNBP1, DLG4, NRXN1, NLGN1, KIF5A, NRXN3 | GO.0048812 |
| 9 | 3 | GO Process | postsynaptic membrane assembly | 3.17E-05 | NRXN2, NRXN1, NLGN1 | GO.0097104 |
| 9 | 3 | GO Process | excitatory synapse assembly | 3.17E-05 | NRXN2, NRXN1, NLGN1 | GO.1904861 |
| 1271 | 12 | GO Process | nervous system process | 3.20E-05 | NRXN2, SHANK1, GRIN1, DLG2, GRIN2A, DLG4, NRXN1, NLGN1, FZD4, NRXN3, SHANK2, GRIN2B | GO.0050877 |
| 101 | 5 | GO Process | neuromuscular process | 3.20E-05 | SHANK1, GRIN1, GRIN2A, DLG4, NRXN1 | GO.0050905 |
| 41 | 4 | GO Process | regulation of glutamate receptor signaling pathway | 3.20E-05 | SHANK1, DLG4, NRXN1, NLGN1 | GO.1900449 |
| 5163 | 23 | GO Process | cellular component organization | 3.27E-05 | GAPDH, KCNJ2, NRXN2, WNT3A, FZD7, SHANK1, SH3D19, MLC1, DTNBP1, ERBB4, DLG1, DMD, UTRN, GRIN1, HMG20A, DLG4, NRXN1, NLGN1, KIF5A, FZD4, NRXN3, SHANK2, GRIN2B | GO.0016043 |
| 18 | 3 | SMART Domains | Eukaryotic homologues of bacterial periplasmic substrate binding proteins. | 3.53E-05 | GRIN1, GRIN2A, GRIN2B | SM00079 |
| 19 | 3 | SMART Domains | Zinc-binding domain, present in Dystrophin, CREB-binding protein. | 3.53E-05 | DMD, UTRN, DTNB | SM00291 |
| 18 | 3 | SMART Domains | Ligated ion channel L-glutamate- and glycine-binding site | 3.53E-05 | GRIN1, GRIN2A, GRIN2B | SM00918 |
| 2788 | 17 | GO Process | regulation of multicellular organismal process | 3.54E-05 | GAPDH, KCNJ2, WNT3A, FZD7, SHANK1, ERBB4, DLG1, DMD, UTRN, GRIN1, HMG20A, GRIN2A, DLG4, NRXN1, NLGN1, FZD4, NOS1 | GO.0051239 |
| 43 | 4 | GO Process | axonal transport | 3.54E-05 | KIF1B, DTNBP1, DLG2, KIF5A | GO.0098930 |
| 17 | 3 | Pfam | Zinc finger, ZZ type | 3.55E-05 | DMD, UTRN, DTNB | PF00569 |
| 11 | 3 | GO Function | guanylate kinase activity | 3.68E-05 | DLG1, DLG2, DLG4 | GO.0004385 |
| 18 | 3 | Pfam | Ligand-gated ion channel | 3.76E-05 | GRIN1, GRIN2A, GRIN2B | PF00060 |
| 18 | 3 | Pfam | Bacterial extracellular solute-binding proteins, family 3 | 3.76E-05 | GRIN1, GRIN2A, GRIN2B | PF00497 |
| 18 | 3 | Pfam | Ligated ion channel L-glutamate- and glycine-binding site | 3.76E-05 | GRIN1, GRIN2A, GRIN2B | PF10613 |
| 16 | 3 | InterPro Domains | Guanylate kinase, conserved site | 3.82E-05 | DLG1, DLG2, DLG4 | IPR020590 |
| 2486 | 16 | GO Process | regulation of cellular protein metabolic process | 4.08E-05 | GAPDH, WNT3A, FZD7, SH3D19, DTNBP1, ERBB4, DLG1, DMD, GRIN1, HMG20A, GRIN2A, DLG4, NRXN1, FZD4, GRIN2B, NOS1 | GO.0032268 |
| 2180 | 15 | GO Process | cellular localization | 4.38E-05 | KIF1B, NRXN2, WNT3A, SHANK1, DTNBP1, DLG1, DMD, GRIN1, DLG2, GRIN2A, DLG4, NRXN1, NLGN1, KIF5A, GRIN2B | GO.0051641 |
| 47 | 4 | GO Process | regulation of neurotransmitter receptor activity | 4.58E-05 | SHANK1, DLG4, NRXN1, NLGN1 | GO.0099601 |
| 50 | 4 | KEGG Pathways | Amyotrophic lateral sclerosis (ALS) | 4.69E-05 | GRIN1, GRIN2A, GRIN2B, NOS1 | hsa05014 |
| 49 | 4 | KEGG Pathways | Cocaine addiction | 4.69E-05 | GRIN1, GRIN2A, DLG4, GRIN2B | hsa05030 |
| 18 | 3 | InterPro Domains | Zinc finger, ZZ-type | 4.89E-05 | DMD, UTRN, DTNB | IPR000433 |
| 18 | 3 | InterPro Domains | Ionotropic glutamate receptor | 4.89E-05 | GRIN1, GRIN2A, GRIN2B | IPR001320 |
| 18 | 3 | InterPro Domains | Ionotropic glutamate receptor, metazoa | 4.89E-05 | GRIN1, GRIN2A, GRIN2B | IPR001508 |
| 18 | 3 | InterPro Domains | Ionotropic glutamate receptor, L-glutamate and glycine-binding domain | 4.89E-05 | GRIN1, GRIN2A, GRIN2B | IPR019594 |
| 221 | 6 | UniProt Keywords | SH3 domain | 5.21E-05 | SHANK1, SH3D19, DLG1, DLG2, DLG4, SHANK2 | KW-0728 |
| 12 | 3 | GO Process | GDP metabolic process | 5.27E-05 | DLG1, DLG2, DLG4 | GO.0046710 |
| 1631 | 13 | GO Process | positive regulation of cell communication | 5.35E-05 | WNT3A, FZD7, SHANK1, DTNBP1, ERBB4, GRIN1, GRIN2A, DLG4, NRXN1, NLGN1, FZD4, SHANK2, NOS1 | GO.0010647 |
| 1638 | 13 | GO Process | positive regulation of signaling | 5.50E-05 | WNT3A, FZD7, SHANK1, DTNBP1, ERBB4, GRIN1, GRIN2A, DLG4, NRXN1, NLGN1, FZD4, SHANK2, NOS1 | GO.0023056 |
| 892 | 10 | GO Process | positive regulation of transport | 5.50E-05 | GAPDH, KCNJ2, WNT3A, MLC1, DTNBP1, DLG1, DMD, GRIN1, NLGN1, NOS1 | GO.0051050 |
| 26 | 3 | SMART Domains | Guanylate kinase homologues. | 6.00E-05 | DLG1, DLG2, DLG4 | SM00072 |
| 2268 | 15 | GO Process | macromolecule localization | 6.41E-05 | KIF1B, NRXN2, WNT3A, SHANK1, MLC1, DLG1, DMD, GRIN1, DLG2, GRIN2A, DLG4, NRXN1, NLGN1, KIF5A, FZD4 | GO.0033036 |
| 273 | 6 | Reactome Pathways | MAPK family signaling cascades | 6.63E-05 | DLG1, GRIN1, DLG2, GRIN2A, DLG4, GRIN2B | HSA-5683057 |
| 22 | 3 | InterPro Domains | Guanylate kinase-like domain | 6.64E-05 | DLG1, DLG2, DLG4 | IPR008144 |
| 1966 | 14 | GO Process | protein localization | 6.68E-05 | KIF1B, NRXN2, WNT3A, SHANK1, MLC1, DLG1, DMD, GRIN1, DLG2, GRIN2A, DLG4, NRXN1, NLGN1, KIF5A | GO.0008104 |
| 3322 | 18 | GO Process | regulation of molecular function | 6.68E-05 | GAPDH, NRXN2, WNT3A, SHANK1, DTNBP1, ERBB4, DLG1, DMD, UTRN, GRIN1, DLG2, GRIN2A, DLG4, NRXN1, NLGN1, FZD4, GRIN2B, NOS1 | GO.0065009 |
| 5459 | 23 | GO Process | positive regulation of biological process | 7.34E-05 | GAPDH, KCNJ2, TIPIN, KIF1B, WNT3A, FZD7, SHANK1, SH3D19, MLC1, DTNBP1, ERBB4, DLG1, DMD, UTRN, GRIN1, GRIN2A, DLG4, NRXN1, NLGN1, FZD4, SHANK2, GRIN2B, NOS1 | GO.0048518 |
| 56 | 4 | GO Process | regulation of sodium ion transmembrane transport | 7.62E-05 | DLG1, DMD, UTRN, NOS1 | GO.1902305 |
| 26 | 3 | Pfam | Guanylate kinase | 8.04E-05 | DLG1, DLG2, DLG4 | PF00625 |
| 2 | 2 | Pfam | L27_1 | 8.04E-05 | DLG1, DLG2 | PF09058 |
| 15 | 3 | GO Process | presynapse assembly | 8.15E-05 | WNT3A, NRXN1, NLGN1 | GO.0099054 |
| 58 | 4 | GO Process | purine ribonucleoside diphosphate metabolic process | 8.52E-05 | GAPDH, DLG1, DLG2, DLG4 | GO.0009179 |
| 16 | 3 | GO Process | retrograde axonal transport | 9.28E-05 | KIF1B, DLG2, KIF5A | GO.0008090 |
| 16 | 3 | GO Process | GMP metabolic process | 9.28E-05 | DLG1, DLG2, DLG4 | GO.0046037 |
| 26 | 3 | InterPro Domains | Guanylate kinase/L-type calcium channel beta subunit | 9.97E-05 | DLG1, DLG2, DLG4 | IPR008145 |
| 2 | 2 | InterPro Domains | Dystrophin/utrophin | 9.97E-05 | DMD, UTRN | IPR035436 |
| 251 | 6 | GO Process | positive regulation of neuron projection development | 1.00E-04 | WNT3A, SHANK1, DMD, DLG4, NLGN1, FZD4 | GO.0010976 |
| 1496 | 12 | GO Process | positive regulation of cellular protein metabolic process | 1.10E-04 | WNT3A, FZD7, SH3D19, ERBB4, DLG1, GRIN1, GRIN2A, DLG4, NRXN1, FZD4, GRIN2B, NOS1 | GO.0032270 |
| 1493 | 12 | GO Process | cell development | 1.10E-04 | WNT3A, FZD7, SHANK1, DTNBP1, ERBB4, DMD, DLG4, NRXN1, NLGN1, KIF5A, FZD4, NRXN3 | GO.0048468 |
| 400 | 7 | GO Process | cell morphogenesis involved in neuron differentiation | 1.10E-04 | WNT3A, SHANK1, DTNBP1, DLG4, NRXN1, KIF5A, NRXN3 | GO.0048667 |
| 3 | 2 | Pfam | N-methyl D-aspartate receptor 2B3 C-terminus | 1.20E-04 | GRIN2A, GRIN2B | PF10565 |
| 19 | 3 | GO Function | extracellularly glutamate-gated ion channel activity | 1.30E-04 | GRIN1, GRIN2A, GRIN2B | GO.0005234 |
| 11878 | 33 | GO Function | binding | 1.30E-04 | GAPDH, KCNJ2, TIPIN, KIF1B, NRXN2, WNT3A, FZD7, SHANK1, SH3D19, MLC1, DLGAP1, ERBB4, DLG1, DMD, UTRN, GRIN1, DLGAP3, DLGAP4, DLG2, HMG20A, MPRIP, GRIN2A, DLG4, DTNB, NRXN1, NLGN1, DLGAP2, KIF5A, FZD4, NRXN3, SHANK2, GRIN2B, NOS1 | GO.0005488 |
| 690 | 8 | GO Component | cell surface | 1.40E-04 | WNT3A, DMD, GRIN1, GRIN2A, NRXN1, NLGN1, FZD4, GRIN2B | GO.0009986 |
| 34 | 3 | Pfam | Laminin G domain | 1.40E-04 | NRXN2, NRXN1, NRXN3 | PF00054 |
| 3 | 2 | InterPro Domains | Glutamate [NMDA] receptor, epsilon subunit, C-terminal | 1.50E-04 | GRIN2A, GRIN2B | IPR018884 |
| 36 | 3 | Pfam | Receptor family ligand binding region | 1.50E-04 | GRIN1, GRIN2A, GRIN2B | PF01094 |
| 4 | 2 | Pfam | N-terminal or F0 domain of Talin-head FERM | 1.50E-04 | SHANK1, SHANK2 | PF16511 |
| 595 | 8 | GO Process | regulation of neuron differentiation | 1.50E-04 | WNT3A, SHANK1, DMD, GRIN1, HMG20A, DLG4, NLGN1, FZD4 | GO.0045664 |
| 3559 | 18 | GO Process | regulation of biological quality | 1.50E-04 | GAPDH, KCNJ2, NRXN2, WNT3A, SHANK1, DTNBP1, ERBB4, DLG1, DMD, GRIN1, GRIN2A, DLG4, NRXN1, NLGN1, FZD4, SHANK2, GRIN2B, NOS1 | GO.0065008 |
| 20 | 3 | GO Process | regulation of NMDA receptor activity | 1.50E-04 | DLG4, NRXN1, NLGN1 | GO.2000310 |
| 20 | 3 | GO Process | regulation of AMPA receptor activity | 1.50E-04 | SHANK1, NRXN1, NLGN1 | GO.2000311 |
| 168 | 5 | KEGG Pathways | Alzheimer's disease | 1.60E-04 | GAPDH, GRIN1, GRIN2A, GRIN2B, NOS1 | hsa05010 |
| 39 | 3 | Pfam | Laminin G domain | 1.70E-04 | NRXN2, NRXN1, NRXN3 | PF02210 |
| 74 | 4 | GO Process | regulation of cardiac muscle contraction | 1.80E-04 | KCNJ2, DLG1, DMD, NOS1 | GO.0055117 |
| 443 | 7 | GO Process | regulation of neuron projection development | 1.90E-04 | WNT3A, SHANK1, DMD, GRIN1, DLG4, NLGN1, FZD4 | GO.0010975 |
| 442 | 7 | GO Process | regulation of cell morphogenesis | 1.90E-04 | WNT3A, SH3D19, DLG1, GRIN1, DLG4, NLGN1, FZD4 | GO.0022604 |
| 2524 | 15 | GO Process | regulation of localization | 1.90E-04 | GAPDH, KCNJ2, WNT3A, SHANK1, MLC1, DTNBP1, ERBB4, DLG1, DMD, UTRN, GRIN1, DLG4, NRXN1, NLGN1, NOS1 | GO.0032879 |
| 1876 | 13 | GO Process | regulation of multicellular organismal development | 1.90E-04 | WNT3A, FZD7, SHANK1, ERBB4, DLG1, DMD, GRIN1, HMG20A, DLG4, NRXN1, NLGN1, FZD4, NOS1 | GO.2000026 |
| 36 | 3 | InterPro Domains | Receptor, ligand binding region | 2.10E-04 | GRIN1, GRIN2A, GRIN2B | IPR001828 |
| 295 | 6 | GO Process | regulation of neurotransmitter levels | 2.20E-04 | NRXN2, DTNBP1, GRIN2A, NRXN1, NLGN1, NOS1 | GO.0001505 |
| 1616 | 12 | GO Process | establishment of localization in cell | 2.20E-04 | KIF1B, NRXN2, WNT3A, DTNBP1, DLG1, GRIN1, DLG2, GRIN2A, NRXN1, NLGN1, KIF5A, GRIN2B | GO.0051649 |
| 44 | 3 | SMART Domains | Laminin G domain | 2.30E-04 | NRXN2, NRXN1, NRXN3 | SM00282 |
| 25 | 3 | GO Process | ionotropic glutamate receptor signaling pathway | 2.50E-04 | GRIN1, GRIN2A, GRIN2B | GO.0035235 |
| 25 | 3 | GO Process | nucleoside monophosphate phosphorylation | 2.50E-04 | DLG1, DLG2, DLG4 | GO.0046940 |
| 39 | 3 | InterPro Domains | Periplasmic binding protein-like I | 2.60E-04 | GRIN1, GRIN2A, GRIN2B | IPR028082 |
| 93 | 4 | KEGG Pathways | Circadian entrainment | 2.60E-04 | GRIN1, GRIN2A, GRIN2B, NOS1 | hsa04713 |
| 2 | 2 | GO Process | positive regulation of presynaptic active zone assembly | 2.70E-04 | NRXN1, NLGN1 | GO.1905520 |
| 89 | 4 | GO Function | PDZ domain binding | 2.80E-04 | FZD7, DLG4, NLGN1, FZD4 | GO.0030165 |
| 8066 | 27 | UniProt Keywords | Phosphoprotein | 2.80E-04 | GAPDH, TIPIN, KIF1B, SHANK1, SH3D19, MLC1, DLGAP1, DTNBP1, ERBB4, DLG1, DMD, UTRN, GRIN1, DLGAP3, DLGAP4, DLG2, HMG20A, MPRIP, GRIN2A, DLG4, DTNB, DLGAP2, KIF5A, BEGAIN, SHANK2, GRIN2B, NOS1 | KW-0597 |
| 27 | 3 | GO Process | startle response | 2.90E-04 | GRIN1, GRIN2A, NRXN1 | GO.0001964 |
| 329 | 6 | GO Function | ion gated channel activity | 3.00E-04 | KCNJ2, DLG1, GRIN1, GRIN2A, DLG4, GRIN2B | GO.0022839 |
| 1992 | 13 | GO Process | anatomical structure morphogenesis | 3.10E-04 | WNT3A, FZD7, SHANK1, DTNBP1, ERBB4, DLG1, DLG4, NRXN1, NLGN1, KIF5A, FZD4, NRXN3, NOS1 | GO.0009653 |
| 3 | 2 | GO Function | GKAP/Homer scaffold activity | 3.20E-04 | SHANK1, SHANK2 | GO.0030160 |
| 200 | 5 | GO Function | cell adhesion molecule binding | 3.20E-04 | NRXN2, UTRN, NRXN1, NLGN1, NRXN3 | GO.0050839 |
| 488 | 7 | GO Process | positive regulation of nervous system development | 3.20E-04 | WNT3A, SHANK1, DMD, DLG4, NRXN1, NLGN1, FZD4 | GO.0051962 |
| 102 | 4 | GO Function | ligand-gated cation channel activity | 3.70E-04 | KCNJ2, GRIN1, GRIN2A, GRIN2B | GO.0099094 |
| 93 | 4 | UniProt Keywords | Alternative promoter usage | 3.70E-04 | NRXN2, UTRN, NRXN1, NRXN3 | KW-0877 |
| 402 | 6 | GO Component | cytoplasmic region | 4.10E-04 | KIF1B, DTNBP1, UTRN, DLG2, DLG4, KIF5A | GO.0099568 |
| 3 | 2 | GO Process | AMPA glutamate receptor clustering | 4.20E-04 | DLG4, NLGN1 | GO.0097113 |
| 3 | 2 | GO Process | gephyrin clustering involved in postsynaptic density assembly | 4.20E-04 | NRXN2, NRXN1 | GO.0097116 |
| 3 | 2 | GO Process | retrograde trans-synaptic signaling | 4.20E-04 | NLGN1, NOS1 | GO.0098917 |
| 250 | 5 | GO Component | distal axon | 4.40E-04 | DTNBP1, UTRN, GRIN1, NRXN1, SHANK2 | GO.0150034 |
| 5 | 2 | NetworkNeighborAL | Dystrophin/utrophin, and nerve maturation | 4.60E-04 | DMD, UTRN | CL.1109 |
| 2416 | 14 | GO Process | regulation of developmental process | 4.70E-04 | WNT3A, FZD7, SHANK1, SH3D19, ERBB4, DLG1, DMD, GRIN1, HMG20A, DLG4, NRXN1, NLGN1, FZD4, NOS1 | GO.0050793 |
| 34 | 3 | GO Process | anterograde axonal transport | 5.10E-04 | KIF1B, DTNBP1, DLG2 | GO.0008089 |
| 40 | 3 | KEGG Pathways | Nicotine addiction | 5.30E-04 | GRIN1, GRIN2A, GRIN2B | hsa05033 |
| 360 | 6 | GO Process | regulation of metal ion transport | 5.40E-04 | KCNJ2, DLG1, DMD, UTRN, GRIN1, NOS1 | GO.0010959 |
| 4 | 2 | GO Process | synaptic vesicle clustering | 6.00E-04 | NRXN1, NLGN1 | GO.0097091 |
| 4 | 2 | GO Process | NMDA glutamate receptor clustering | 6.00E-04 | NRXN1, NLGN1 | GO.0097114 |
| 4 | 2 | GO Process | neuroligin clustering involved in postsynaptic membrane assembly | 6.00E-04 | NRXN2, NRXN1 | GO.0097118 |
| 11 | 2 | SMART Domains | Frizzled/Smoothened family membrane region | 6.20E-04 | FZD7, FZD4 | SM01330 |
| 224 | 5 | GO Process | regulation of muscle system process | 6.30E-04 | KCNJ2, DLG1, DMD, UTRN, NOS1 | GO.0090257 |
| 4898 | 20 | GO Process | positive regulation of cellular process | 6.50E-04 | GAPDH, KCNJ2, TIPIN, WNT3A, FZD7, SHANK1, SH3D19, DTNBP1, ERBB4, DLG1, DMD, GRIN1, GRIN2A, DLG4, NRXN1, NLGN1, FZD4, SHANK2, GRIN2B, NOS1 | GO.0048522 |
| 4454 | 19 | GO Process | negative regulation of cellular process | 6.50E-04 | GAPDH, TIPIN, WNT3A, FZD7, SHANK1, DTNBP1, ERBB4, DLG1, DMD, GRIN1, DLG2, HMG20A, DLG4, NRXN1, NLGN1, FZD4, SHANK2, GRIN2B, NOS1 | GO.0048523 |
| 226 | 5 | GO Process | vesicle localization | 6.50E-04 | KIF1B, DTNBP1, NRXN1, NLGN1, KIF5A | GO.0051648 |
| 243 | 5 | GO Function | Ras guanyl-nucleotide exchange factor activity | 6.70E-04 | ERBB4, GRIN1, GRIN2A, DLG4, GRIN2B | GO.0005088 |
| 1551 | 11 | GO Process | positive regulation of multicellular organismal process | 6.70E-04 | GAPDH, WNT3A, SHANK1, ERBB4, DMD, GRIN1, DLG4, NRXN1, NLGN1, FZD4, NOS1 | GO.0051240 |
| 383 | 6 | GO Process | cell surface receptor signaling pathway involved in cell-cell signaling | 7.00E-04 | WNT3A, FZD7, GRIN1, GRIN2A, FZD4, GRIN2B | GO.1905114 |
| 40 | 3 | GO Process | long-term synaptic potentiation | 7.30E-04 | GRIN2A, NLGN1, SHANK2 | GO.0060291 |
| 4953 | 20 | GO Process | negative regulation of biological process | 7.50E-04 | GAPDH, TIPIN, WNT3A, FZD7, SHANK1, DTNBP1, ERBB4, DLG1, DMD, GRIN1, DLG2, HMG20A, GRIN2A, DLG4, NRXN1, NLGN1, FZD4, SHANK2, GRIN2B, NOS1 | GO.0048519 |
| 58 | 3 | InterPro Domains | Laminin G domain | 7.60E-04 | NRXN2, NRXN1, NRXN3 | IPR001791 |
| 41 | 3 | GO Process | vesicle transport along microtubule | 7.60E-04 | KIF1B, DTNBP1, KIF5A | GO.0047496 |
| 5 | 2 | GO Process | synaptic membrane adhesion | 7.60E-04 | NRXN1, NLGN1 | GO.0099560 |
| 5 | 2 | GO Process | axo-dendritic protein transport | 7.60E-04 | DLG2, KIF5A | GO.0099640 |
| 1034 | 9 | GO Process | plasma membrane bounded cell projection organization | 7.60E-04 | WNT3A, SHANK1, DTNBP1, DMD, DLG4, NRXN1, NLGN1, KIF5A, NRXN3 | GO.0120036 |
| 5 | 2 | GO Process | retrograde neuronal dense core vesicle transport | 7.60E-04 | KIF1B, KIF5A | GO.1990049 |
| 5 | 2 | GO Process | regulation of grooming behavior | 7.60E-04 | DLG4, NRXN1 | GO.2000821 |
| 7 | 2 | Reactome Pathways | NrCAM interactions | 7.60E-04 | DLG1, DLG4 | HSA-447038 |
| 4792 | 19 | GO Component | protein-containing complex | 7.80E-04 | GAPDH, KCNJ2, TIPIN, KIF1B, WNT3A, SHANK1, DTNBP1, ERBB4, DLG1, DMD, UTRN, GRIN1, GRIN2A, DLG4, NRXN1, NLGN1, KIF5A, GRIN2B, NOS1 | GO.0032991 |
| 12 | 2 | Pfam | Frizzled/Smoothened family membrane region | 8.10E-04 | FZD7, FZD4 | PF01534 |
| 244 | 5 | GO Process | muscle contraction | 8.50E-04 | KCNJ2, DLG1, DMD, UTRN, NOS1 | GO.0006936 |
| 3327 | 16 | GO Process | regulation of cell communication | 8.50E-04 | WNT3A, FZD7, SHANK1, DTNBP1, ERBB4, DLG1, DMD, GRIN1, GRIN2A, DLG4, NRXN1, NLGN1, FZD4, SHANK2, GRIN2B, NOS1 | GO.0010646 |
| 2249 | 13 | GO Process | regulation of catalytic activity | 8.70E-04 | GAPDH, WNT3A, DTNBP1, ERBB4, DLG1, GRIN1, DLG2, GRIN2A, DLG4, NRXN1, FZD4, GRIN2B, NOS1 | GO.0050790 |
| 4153 | 18 | GO Process | response to chemical | 8.80E-04 | GAPDH, KIF1B, WNT3A, FZD7, SHANK1, MLC1, ERBB4, GRIN1, DLG2, GRIN2A, DLG4, NRXN1, NLGN1, KIF5A, FZD4, NRXN3, GRIN2B, NOS1 | GO.0042221 |
| 139 | 4 | KEGG Pathways | Cell adhesion molecules (CAMs) | 8.90E-04 | NRXN2, NRXN1, NLGN1, NRXN3 | hsa04514 |
| 8 | 2 | Reactome Pathways | RNF mutants show enhanced WNT signaling and proliferation | 8.90E-04 | WNT3A, FZD4 | HSA-5340588 |
| 155 | 4 | GO Component | synaptic vesicle | 9.10E-04 | DTNBP1, GRIN1, GRIN2A, DLG4 | GO.0008021 |
| 137 | 4 | GO Function | channel regulator activity | 9.10E-04 | NRXN2, DLG1, NRXN1, NOS1 | GO.0016247 |
| 434 | 6 | GO Function | substrate-specific channel activity | 9.10E-04 | KCNJ2, DLG1, GRIN1, GRIN2A, DLG4, GRIN2B | GO.0022838 |
| 142 | 4 | GO Function | voltage-gated cation channel activity | 9.10E-04 | KCNJ2, GRIN1, GRIN2A, GRIN2B | GO.0022843 |
| 6 | 2 | GO Process | negative regulation of cell fate specification | 9.30E-04 | WNT3A, FZD7 | GO.0009996 |
| 6 | 2 | GO Process | peptidyl-cysteine S-nitrosylation | 9.30E-04 | GAPDH, NOS1 | GO.0018119 |
| 3360 | 16 | GO Process | regulation of signaling | 9.30E-04 | WNT3A, FZD7, SHANK1, DTNBP1, ERBB4, DLG1, DMD, GRIN1, GRIN2A, DLG4, NRXN1, NLGN1, FZD4, SHANK2, GRIN2B, NOS1 | GO.0023051 |
| 6507 | 23 | GO Process | multicellular organismal process | 9.30E-04 | KCNJ2, NRXN2, WNT3A, FZD7, SHANK1, DTNBP1, ERBB4, DLG1, DMD, UTRN, GRIN1, DLG2, HMG20A, GRIN2A, DLG4, NRXN1, NLGN1, KIF5A, FZD4, NRXN3, SHANK2, GRIN2B, NOS1 | GO.0032501 |
| 6 | 2 | GO Process | vocal learning | 9.30E-04 | NRXN2, NRXN1 | GO.0042297 |
| 252 | 5 | GO Process | positive regulation of ion transport | 9.30E-04 | KCNJ2, DLG1, DMD, GRIN1, NOS1 | GO.0043270 |
| 416 | 6 | GO Process | cell-cell adhesion | 9.30E-04 | NRXN2, WNT3A, DLG1, NRXN1, NLGN1, NRXN3 | GO.0098609 |
| 1355 | 10 | GO Process | movement of cell or subcellular component | 9.50E-04 | KCNJ2, KIF1B, WNT3A, DTNBP1, ERBB4, DMD, DLG2, NRXN1, KIF5A, NRXN3 | GO.0006928 |
| 159 | 4 | GO Component | growth cone | 9.80E-04 | DTNBP1, UTRN, NRXN1, SHANK2 | GO.0030426 |
| 132 | 4 | GO Process | calcium-mediated signaling | 9.80E-04 | DMD, GRIN1, GRIN2A, GRIN2B | GO.0019722 |
| 10 | 2 | GO Component | juxtaparanode region of axon | 9.90E-04 | DLG2, DLG4 | GO.0044224 |
| 10 | 2 | GO Component | integral component of postsynaptic membrane | 9.90E-04 | GRIN2A, NLGN1 | GO.0099055 |
| 11 | 2 | InterPro Domains | Frizzled/Smoothened, transmembrane domain | 9.90E-04 | FZD7, FZD4 | IPR000539 |
| 843 | 8 | GO Process | cell adhesion | 9.90E-04 | NRXN2, WNT3A, FZD7, DLG1, NRXN1, NLGN1, FZD4, NRXN3 | GO.0007155 |
| 1657 | 11 | GO Process | regulation of phosphate metabolic process | 9.90E-04 | WNT3A, FZD7, DTNBP1, ERBB4, DLG1, DMD, DLG2, DLG4, NRXN1, FZD4, NOS1 | GO.0019220 |
| 57 | 3 | GO Function | amyloid-beta binding | 0.001 | DLGAP3, NLGN1, FZD4 | GO.0001540 |
| 57 | 3 | GO Function | amino acid binding | 0.001 | GRIN1, GRIN2B, NOS1 | GO.0016597 |
| 1429 | 10 | GO Function | signaling receptor activity | 0.001 | NRXN2, FZD7, ERBB4, GRIN1, GRIN2A, NRXN1, NLGN1, FZD4, NRXN3, GRIN2B | GO.0038023 |
| 49 | 3 | GO Process | neuromuscular process controlling balance | 0.001 | SHANK1, DLG4, NRXN1 | GO.0050885 |
| 7 | 2 | GO Process | neuron-neuron synaptic transmission | 0.0011 | KIF1B, DLGAP2 | GO.0007270 |
| 266 | 5 | GO Process | second-messenger-mediated signaling | 0.0011 | DMD, GRIN1, GRIN2A, GRIN2B, NOS1 | GO.0019932 |
| 2343 | 13 | GO Process | cellular component assembly | 0.0011 | KCNJ2, NRXN2, WNT3A, SHANK1, MLC1, DLG1, DMD, GRIN1, DLG4, NRXN1, NLGN1, SHANK2, GRIN2B | GO.0022607 |
| 7 | 2 | GO Process | neuronal signal transduction | 0.0011 | NRXN1, NLGN1 | GO.0023041 |
| 1381 | 10 | GO Process | positive regulation of catalytic activity | 0.0011 | WNT3A, ERBB4, DLG1, GRIN1, GRIN2A, DLG4, NRXN1, FZD4, GRIN2B, NOS1 | GO.0043085 |
| 1695 | 11 | GO Process | regulation of cell differentiation | 0.0011 | WNT3A, FZD7, SHANK1, ERBB4, DMD, GRIN1, HMG20A, DLG4, NLGN1, FZD4, NOS1 | GO.0045595 |
| 7 | 2 | GO Process | positive regulation of neuron projection arborization | 0.0011 | DLG4, FZD4 | GO.0150012 |
| 139 | 4 | GO Process | positive regulation of cysteine-type endopeptidase activity | 0.0011 | WNT3A, GRIN1, GRIN2A, GRIN2B | GO.2001056 |
| 67 | 3 | GO Component | main axon | 0.0012 | DLG1, DLG2, DLG4 | GO.0044304 |
| 10 | 2 | GO Function | vinculin binding | 0.0012 | DMD, UTRN | GO.0017166 |
| 1793 | 11 | GO Function | molecular function regulator | 0.0012 | GAPDH, NRXN2, WNT3A, ERBB4, DLG1, GRIN1, GRIN2A, DLG4, NRXN1, GRIN2B, NOS1 | GO.0098772 |
| 5219 | 20 | GO Process | cell communication | 0.0012 | KIF1B, NRXN2, WNT3A, FZD7, DLGAP1, ERBB4, DLG1, DMD, GRIN1, DLG2, GRIN2A, DLG4, NRXN1, NLGN1, DLGAP2, KIF5A, FZD4, NRXN3, GRIN2B, NOS1 | GO.0007154 |
| 650 | 7 | GO Process | brain development | 0.0012 | WNT3A, ERBB4, GRIN1, GRIN2A, NRXN1, FZD4, GRIN2B | GO.0007420 |
| 8 | 2 | GO Process | olfactory behavior | 0.0012 | SHANK1, GRIN1 | GO.0042048 |
| 1713 | 11 | GO Process | positive regulation of molecular function | 0.0012 | WNT3A, ERBB4, DLG1, DMD, GRIN1, GRIN2A, DLG4, NRXN1, FZD4, GRIN2B, NOS1 | GO.0044093 |
| 54 | 3 | GO Process | dendrite morphogenesis | 0.0012 | SHANK1, DTNBP1, DLG4 | GO.0048813 |
| 1128 | 9 | GO Process | positive regulation of cellular component organization | 0.0012 | WNT3A, SHANK1, DLG1, DMD, DLG4, NRXN1, NLGN1, FZD4, NOS1 | GO.0051130 |
| 52 | 3 | GO Process | protein heterotetramerization | 0.0012 | GRIN1, NLGN1, GRIN2B | GO.0051290 |
| 53 | 3 | GO Process | positive regulation of protein localization to plasma membrane | 0.0012 | WNT3A, DLG1, NRXN1 | GO.1903078 |
| 10 | 2 | Reactome Pathways | Ionotropic activity of kainate receptors | 0.0012 | DLG1, DLG4 | HSA-451306 |
| 10 | 2 | Reactome Pathways | Activation of Ca-permeable Kainate Receptor | 0.0012 | DLG1, DLG4 | HSA-451308 |
| 1226 | 9 | GO Function | transmembrane signaling receptor activity | 0.0013 | NRXN2, FZD7, ERBB4, GRIN1, GRIN2A, NRXN1, FZD4, NRXN3, GRIN2B | GO.0004888 |
| 11 | 2 | GO Function | glutamate binding | 0.0013 | GRIN1, GRIN2B | GO.0016595 |
| 11 | 2 | GO Function | acetylcholine receptor binding | 0.0013 | DLG4, NRXN1 | GO.0033130 |
| 10 | 2 | NetworkNeighborAL | retrograde neuronal dense core vesicle transport, and Kinesin light chain | 0.0013 | KIF1B, KIF5A | CL.18531 |
| 1564 | 10 | GO Component | integral component of plasma membrane | 0.0014 | KCNJ2, SHANK1, GRIN1, GRIN2A, DLG4, NRXN1, NLGN1, FZD4, NRXN3, GRIN2B | GO.0005887 |
| 68 | 3 | GO Function | protein-containing complex scaffold activity | 0.0014 | SHANK1, DLG1, SHANK2 | GO.0032947 |
| 1747 | 11 | GO Process | regulation of protein modification process | 0.0014 | WNT3A, FZD7, DTNBP1, ERBB4, DLG1, DMD, HMG20A, DLG4, NRXN1, FZD4, NOS1 | GO.0031399 |
| 7824 | 25 | GO Process | response to stimulus | 0.0014 | GAPDH, KCNJ2, TIPIN, KIF1B, NRXN2, WNT3A, FZD7, SHANK1, MLC1, DTNBP1, ERBB4, DLG1, DMD, UTRN, GRIN1, DLG2, GRIN2A, DLG4, NRXN1, NLGN1, KIF5A, FZD4, NRXN3, GRIN2B, NOS1 | GO.0050896 |
| 9 | 2 | GO Process | synapse maturation | 0.0014 | SHANK1, NRXN1 | GO.0060074 |
| 287 | 5 | GO Process | divalent metal ion transport | 0.0014 | KCNJ2, WNT3A, GRIN1, GRIN2A, GRIN2B | GO.0070838 |
| 9 | 2 | GO Process | presynaptic membrane assembly | 0.0014 | NRXN1, NLGN1 | GO.0097105 |
| 58 | 3 | GO Process | calcium ion transmembrane import into cytosol | 0.0014 | GRIN1, GRIN2A, GRIN2B | GO.0097553 |
| 18 | 2 | SMART Domains | Frizzled | 0.0014 | FZD7, FZD4 | SM00063 |
| 179 | 4 | KEGG Pathways | Calcium signaling pathway | 0.0015 | ERBB4, GRIN1, GRIN2A, NOS1 | hsa04020 |
| 64 | 3 | KEGG Pathways | Long-term potentiation | 0.0015 | GRIN1, GRIN2A, GRIN2B | hsa04720 |
| 65 | 3 | KEGG Pathways | Amphetamine addiction | 0.0015 | GRIN1, GRIN2A, GRIN2B | hsa05031 |
| 317 | 5 | KEGG Pathways | Human papillomavirus infection | 0.0015 | WNT3A, FZD7, DLG1, DLG2, FZD4 | hsa05165 |
| 63 | 3 | KEGG Pathways | Basal cell carcinoma | 0.0015 | WNT3A, FZD7, FZD4 | hsa05217 |
| 866 | 8 | UniProt Keywords | Calcium | 0.0015 | NRXN2, DMD, UTRN, GRIN1, GRIN2A, NRXN1, NRXN3, GRIN2B | KW-0106 |
| 157 | 4 | GO Process | protein tetramerization | 0.0015 | KCNJ2, GRIN1, NLGN1, GRIN2B | GO.0051262 |
| 10 | 2 | GO Process | cellular response to potassium ion | 0.0016 | DLG2, DLG4 | GO.0035865 |
| 161 | 4 | GO Process | regulation of synapse organization | 0.0016 | GRIN1, NRXN1, NLGN1, GRIN2B | GO.0050807 |
| 1493 | 10 | GO Process | positive regulation of signal transduction | 0.0017 | WNT3A, FZD7, SHANK1, ERBB4, GRIN1, DLG4, NRXN1, NLGN1, FZD4, NOS1 | GO.0009967 |
| 163 | 4 | GO Process | negative regulation of cell projection organization | 0.0017 | WNT3A, NRXN1, NLGN1, GRIN2B | GO.0031345 |
| 162 | 4 | GO Process | cellular response to metal ion | 0.0017 | DLG2, DLG4, NRXN1, NLGN1 | GO.0071248 |
| 14 | 2 | GO Function | glycine binding | 0.0018 | GRIN1, GRIN2B | GO.0016594 |
| 195 | 4 | KEGG Pathways | Proteoglycans in cancer | 0.0018 | WNT3A, FZD7, ERBB4, FZD4 | hsa05205 |
| 165 | 4 | GO Process | signal release | 0.0018 | NRXN2, NRXN1, NLGN1, FZD4 | GO.0023061 |
| 1514 | 10 | GO Process | protein-containing complex assembly | 0.0018 | KCNJ2, WNT3A, SHANK1, MLC1, DMD, GRIN1, DLG4, NRXN1, NLGN1, GRIN2B | GO.0065003 |
| 16 | 2 | InterPro Domains | Frizzled/secreted frizzled-related protein | 0.0019 | FZD7, FZD4 | IPR015526 |
| 961 | 8 | GO Process | regulation of anatomical structure morphogenesis | 0.0019 | WNT3A, FZD7, SH3D19, DLG1, GRIN1, DLG4, NLGN1, FZD4 | GO.0022603 |
| 72 | 3 | NetworkNeighborAL | Wnt signaling pathway, and coreceptor activity involved in Wnt signaling pathway, planar cell polarity pathway | 0.002 | WNT3A, FZD7, FZD4 | CL.5701 |
| 323 | 5 | GO Process | MAPK cascade | 0.0021 | ERBB4, GRIN1, GRIN2A, DLG4, GRIN2B | GO.0000165 |
| 4130 | 17 | GO Process | transport | 0.0021 | KCNJ2, KIF1B, NRXN2, WNT3A, MLC1, DTNBP1, ERBB4, DLG1, GRIN1, DLG2, GRIN2A, DLG4, NRXN1, NLGN1, KIF5A, FZD4, GRIN2B | GO.0006810 |
| 1857 | 11 | GO Process | response to external stimulus | 0.0021 | GAPDH, KCNJ2, NRXN2, WNT3A, SHANK1, DMD, GRIN1, GRIN2A, NRXN1, KIF5A, NRXN3 | GO.0009605 |
| 12 | 2 | GO Process | regulation of skeletal muscle contraction | 0.0021 | KCNJ2, DMD | GO.0014819 |
| 68 | 3 | GO Process | regulation of behavior | 0.0021 | DLG4, NRXN1, NLGN1 | GO.0050795 |
| 729 | 7 | GO Process | membrane organization | 0.0021 | NRXN2, DLG1, DLG4, NRXN1, NLGN1, KIF5A, FZD4 | GO.0061024 |
| 68 | 3 | GO Process | cellular response to retinoic acid | 0.0021 | WNT3A, FZD7, FZD4 | GO.0071300 |
| 17 | 2 | GO Component | filopodium membrane | 0.0022 | DMD, UTRN | GO.0031527 |
| 22 | 2 | Pfam | Spectrin repeat | 0.0023 | DMD, UTRN | PF00435 |
| 181 | 4 | GO Process | protein localization to cell periphery | 0.0023 | KIF1B, DLG1, GRIN1, GRIN2A | GO.1990778 |
| 23 | 2 | Pfam | Fz domain | 0.0024 | FZD7, FZD4 | PF01392 |
| 73 | 3 | GO Process | regulation of cardiac muscle tissue development | 0.0024 | WNT3A, FZD7, ERBB4 | GO.0055024 |
| 213 | 4 | GO Component | basolateral plasma membrane | 0.0025 | MLC1, ERBB4, DLG1, DLG4 | GO.0016323 |
| 75 | 3 | GO Process | associative learning | 0.0026 | SHANK1, GRIN1, GRIN2A | GO.0008306 |
| 75 | 3 | GO Process | sensory perception of pain | 0.0026 | GRIN1, DLG2, GRIN2A | GO.0019233 |
| 347 | 5 | GO Process | positive regulation of proteolysis | 0.0026 | WNT3A, SH3D19, GRIN1, GRIN2A, GRIN2B | GO.0045862 |
| 76 | 3 | GO Process | excitatory postsynaptic potential | 0.0026 | GRIN1, GRIN2A, GRIN2B | GO.0060079 |
| 14 | 2 | GO Process | prepulse inhibition | 0.0026 | GRIN1, NRXN1 | GO.0060134 |
| 189 | 4 | GO Process | calcium ion transmembrane transport | 0.0026 | WNT3A, GRIN1, GRIN2A, GRIN2B | GO.0070588 |
| 16 | 2 | Reactome Pathways | MECP2 regulates neuronal receptors and channels | 0.0026 | GRIN2A, GRIN2B | HSA-9022699 |
| 83 | 3 | NetworkNeighborAL | mixed, incl. frizzled binding, and Wnt-protein binding | 0.0028 | WNT3A, FZD7, FZD4 | CL.5700 |
| 15 | 2 | GO Process | protein localization to postsynaptic membrane | 0.0028 | GRIN1, GRIN2A | GO.1903539 |
| 2068 | 11 | GO Component | cytoskeleton | 0.0029 | GAPDH, KIF1B, DTNBP1, DLG1, DMD, UTRN, MPRIP, DLG4, DLGAP2, KIF5A, NOS1 | GO.0005856 |
| 17 | 2 | NetworkNeighborAL | mixed, incl. Laminin IV, and Dystrophin/utrophin | 0.0029 | DMD, UTRN | CL.1085 |
| 553 | 6 | GO Process | positive regulation of kinase activity | 0.0029 | WNT3A, ERBB4, DLG1, DLG4, NRXN1, FZD4 | GO.0033674 |
| 96 | 3 | GO Component | filopodium | 0.003 | DMD, UTRN, NLGN1 | GO.0030175 |
| 2306 | 12 | GO Process | regulation of cellular component organization | 0.003 | WNT3A, SHANK1, SH3D19, DLG1, DMD, GRIN1, DLG4, NRXN1, NLGN1, FZD4, GRIN2B, NOS1 | GO.0051128 |
| 2672 | 13 | GO Process | cellular response to chemical stimulus | 0.003 | GAPDH, KIF1B, WNT3A, FZD7, MLC1, ERBB4, GRIN1, DLG2, DLG4, NRXN1, NLGN1, FZD4, NOS1 | GO.0070887 |
| 21 | 2 | GO Component | dystrophin-associated glycoprotein complex | 0.0031 | DMD, UTRN | GO.0016010 |
| 98 | 3 | KEGG Pathways | Melanogenesis | 0.0031 | WNT3A, FZD7, FZD4 | hsa04916 |
| 16 | 2 | GO Process | positive regulation of neurotransmitter secretion | 0.0031 | DTNBP1, NLGN1 | GO.0001956 |
| 1052 | 8 | GO Process | response to abiotic stimulus | 0.0031 | KCNJ2, TIPIN, NRXN2, DMD, GRIN1, GRIN2A, NRXN1, NOS1 | GO.0009628 |
| 1052 | 8 | GO Process | positive regulation of phosphate metabolic process | 0.0031 | WNT3A, FZD7, ERBB4, DLG1, DLG4, NRXN1, FZD4, NOS1 | GO.0045937 |
| 16 | 2 | GO Process | dendritic spine morphogenesis | 0.0031 | SHANK1, DLG4 | GO.0060997 |
| 22 | 2 | InterPro Domains | Spectrin repeat | 0.0032 | DMD, UTRN | IPR002017 |
| 3081 | 14 | GO Process | positive regulation of macromolecule metabolic process | 0.0033 | KIF1B, WNT3A, FZD7, SH3D19, DTNBP1, ERBB4, DLG1, GRIN1, GRIN2A, DLG4, NRXN1, FZD4, GRIN2B, NOS1 | GO.0010604 |
| 806 | 7 | GO Process | cellular homeostasis | 0.0033 | KCNJ2, DMD, GRIN1, GRIN2A, DLG4, GRIN2B, NOS1 | GO.0019725 |
| 5233 | 19 | GO Process | localization | 0.0033 | KCNJ2, KIF1B, NRXN2, WNT3A, SHANK1, MLC1, DTNBP1, ERBB4, DLG1, DMD, GRIN1, DLG2, GRIN2A, DLG4, NRXN1, NLGN1, KIF5A, FZD4, GRIN2B | GO.0051179 |
| 574 | 6 | GO Process | organelle localization | 0.0033 | KIF1B, DTNBP1, DLG1, NRXN1, NLGN1, KIF5A | GO.0051640 |
| 23 | 2 | InterPro Domains | Actinin-type actin-binding domain, conserved site | 0.0034 | DMD, UTRN | IPR001589 |
| 23 | 2 | InterPro Domains | Frizzled domain | 0.0034 | FZD7, FZD4 | IPR020067 |
| 23 | 2 | InterPro Domains | Frizzled cysteine-rich domain superfamily | 0.0034 | FZD7, FZD4 | IPR036790 |
| 208 | 4 | GO Process | regulation of stress-activated MAPK cascade | 0.0034 | FZD7, DTNBP1, DLG1, FZD4 | GO.0032872 |
| 86 | 3 | GO Process | regulation of synapse assembly | 0.0034 | GRIN1, NRXN1, NLGN1 | GO.0051963 |
| 17 | 2 | GO Process | positive regulation of sodium ion transmembrane transport | 0.0034 | DMD, NOS1 | GO.1902307 |
| 21 | 2 | GO Function | Wnt-activated receptor activity | 0.0035 | FZD7, FZD4 | GO.0042813 |
| 1370 | 9 | GO Process | regulation of protein phosphorylation | 0.0035 | WNT3A, FZD7, DTNBP1, ERBB4, DLG1, DMD, DLG4, NRXN1, FZD4 | GO.0001932 |
| 210 | 4 | GO Process | response to mechanical stimulus | 0.0035 | KCNJ2, NRXN2, DMD, NRXN1 | GO.0009612 |
| 93 | 3 | Reactome Pathways | Class B/2 (Secretin family receptors) | 0.0035 | WNT3A, FZD7, FZD4 | HSA-373080 |
| 1796 | 10 | GO Component | endoplasmic reticulum | 0.0036 | KCNJ2, WNT3A, MLC1, DTNBP1, DLG1, GRIN1, GRIN2A, DLG4, NRXN1, NOS1 | GO.0005783 |
| 100 | 3 | GO Function | ion channel regulator activity | 0.0036 | NRXN2, DLG1, NRXN1 | GO.0099106 |
| 18 | 2 | GO Process | negative regulation of cardiocyte differentiation | 0.0036 | WNT3A, FZD7 | GO.1905208 |
| 882 | 7 | GO Function | cytoskeletal protein binding | 0.0037 | GAPDH, KIF1B, DLG1, DMD, UTRN, MPRIP, KIF5A | GO.0008092 |
| 216 | 4 | GO Process | developmental maturation | 0.0038 | SHANK1, GRIN1, DLG4, NRXN1 | GO.0021700 |
| 387 | 5 | GO Process | establishment of organelle localization | 0.0038 | KIF1B, DTNBP1, DLG1, NLGN1, KIF5A | GO.0051656 |
| 21 | 2 | Reactome Pathways | Regulation of FZD by ubiquitination | 0.0038 | WNT3A, FZD4 | HSA-4641263 |
| 33 | 2 | SMART Domains | Spectrin repeats | 0.0038 | DMD, UTRN | SM00150 |
| 250 | 4 | KEGG Pathways | HTLV-I infection | 0.0039 | WNT3A, FZD7, DLG1, FZD4 | hsa05166 |
| 218 | 4 | GO Process | establishment of vesicle localization | 0.0039 | KIF1B, DTNBP1, NLGN1, KIF5A | GO.0051650 |
| 21 | 2 | NetworkNeighborAL | dystrophin-associated glycoprotein complex, and Distrobrevin | 0.004 | DTNB, NOS1 | CL.2140 |
| 220 | 4 | GO Process | axon guidance | 0.004 | WNT3A, NRXN1, KIF5A, NRXN3 | GO.0007411 |
| 92 | 3 | GO Process | regulation of potassium ion transport | 0.004 | KCNJ2, DLG1, NOS1 | GO.0043266 |
| 92 | 3 | GO Process | regulation of neurotransmitter transport | 0.004 | DTNBP1, NLGN1, NOS1 | GO.0051588 |
| 393 | 5 | GO Process | regulation of endopeptidase activity | 0.004 | GAPDH, WNT3A, GRIN1, GRIN2A, GRIN2B | GO.0052548 |
| 93 | 3 | GO Process | canonical Wnt signaling pathway | 0.004 | WNT3A, FZD7, FZD4 | GO.0060070 |
| 92 | 3 | GO Process | synaptic vesicle localization | 0.004 | DTNBP1, NRXN1, NLGN1 | GO.0097479 |
| 605 | 6 | GO Process | microtubule-based process | 0.0041 | GAPDH, KIF1B, DTNBP1, DLG1, DLG2, KIF5A | GO.0007017 |
| 849 | 7 | GO Process | regulation of kinase activity | 0.0041 | WNT3A, DTNBP1, ERBB4, DLG1, DLG4, NRXN1, FZD4 | GO.0043549 |
| 345 | 5 | UniProt Keywords | Ion channel | 0.0042 | KCNJ2, MLC1, GRIN1, GRIN2A, GRIN2B | KW-0407 |
| 95 | 3 | GO Process | neurotransmitter secretion | 0.0042 | NRXN2, NRXN1, NLGN1 | GO.0007269 |
| 79 | 3 | UniProt Keywords | Ligand-gated ion channel | 0.0043 | GRIN1, GRIN2A, GRIN2B | KW-1071 |
| 26 | 2 | GO Component | dendrite cytoplasm | 0.0044 | DLG4, KIF5A | GO.0032839 |
| 97 | 3 | GO Process | regulation of calcium ion transport into cytosol | 0.0044 | DMD, GRIN1, NOS1 | GO.0010522 |
| 115 | 3 | GO Component | neuron projection terminus | 0.0046 | DMD, GRIN1, DLG4 | GO.0044306 |
| 230 | 4 | GO Process | purine ribonucleoside monophosphate metabolic process | 0.0046 | GAPDH, DLG1, DLG2, DLG4 | GO.0009167 |
| 29 | 2 | InterPro Domains | Spectrin/alpha-actinin | 0.0047 | DMD, UTRN | IPR018159 |
| 1149 | 8 | GO Process | positive regulation of protein modification process | 0.0047 | WNT3A, FZD7, ERBB4, DLG1, DLG4, NRXN1, FZD4, NOS1 | GO.0031401 |
| 233 | 4 | GO Process | response to alcohol | 0.0047 | MLC1, GRIN1, GRIN2A, GRIN2B | GO.0097305 |
| 100 | 3 | GO Process | synaptic vesicle cycle | 0.0047 | WNT3A, NRXN1, NLGN1 | GO.0099504 |
| 2197 | 11 | GO Function | enzyme binding | 0.0048 | KIF1B, ERBB4, DLG1, DMD, UTRN, GRIN1, DLG2, GRIN2A, DLG4, FZD4, GRIN2B | GO.0019899 |
| 632 | 6 | GO Process | negative regulation of cellular component organization | 0.0048 | WNT3A, SHANK1, DLG4, NRXN1, NLGN1, GRIN2B | GO.0051129 |
| 22 | 2 | GO Process | positive regulation of synaptic transmission, glutamatergic | 0.0048 | NRXN1, NLGN1 | GO.0051968 |
| 22 | 2 | GO Process | negative regulation of synapse organization | 0.0048 | NLGN1, GRIN2B | GO.1905809 |
| 17 | 2 | UniProt Keywords | Schizophrenia | 0.0049 | DTNBP1, NRXN1 | KW-1211 |
| 1554 | 9 | GO Component | whole membrane | 0.005 | WNT3A, FZD7, MLC1, DTNBP1, DLG1, DMD, DLG4, FZD4, NOS1 | GO.0098805 |
| 104 | 3 | GO Process | metencephalon development | 0.0051 | GRIN1, NRXN1, FZD4 | GO.0022037 |
| 23 | 2 | GO Process | regulation of respiratory gaseous exchange | 0.0051 | GRIN1, NLGN1 | GO.0043576 |
| 23 | 2 | GO Process | somatic stem cell division | 0.0051 | WNT3A, FZD7 | GO.0048103 |
| 23 | 2 | GO Process | secondary palate development | 0.0051 | WNT3A, DLG1 | GO.0062009 |
| 112 | 3 | NetworkNeighborAL | Wnt signaling pathway, and PET domain | 0.0053 | WNT3A, FZD7, FZD4 | CL.5699 |
| 24 | 2 | GO Process | regulation of long-term neuronal synaptic plasticity | 0.0054 | GRIN1, DLG4 | GO.0048169 |
| 275 | 4 | GO Component | endocytic vesicle | 0.0055 | WNT3A, DLG4, NRXN1, FZD4 | GO.0030139 |
| 16244 | 35 | GO Component | cell part | 0.0055 | GAPDH, KCNJ2, TIPIN, KIF1B, NRXN2, WNT3A, FZD7, SHANK1, SH3D19, MLC1, DLGAP1, DTNBP1, ERBB4, DLG1, DMD, UTRN, GRIN1, DLGAP3, DLGAP4, DLG2, HMG20A, MPRIP, GRIN2A, DLG4, DTNB, NRXN1, NLGN1, DLGAP2, KIF5A, BEGAIN, FZD4, NRXN3, SHANK2, GRIN2B, NOS1 | GO.0044464 |
| 37 | 2 | Pfam | WW domain | 0.0056 | DMD, UTRN | PF00397 |
| 16271 | 35 | GO Component | cell | 0.0057 | GAPDH, KCNJ2, TIPIN, KIF1B, NRXN2, WNT3A, FZD7, SHANK1, SH3D19, MLC1, DLGAP1, DTNBP1, ERBB4, DLG1, DMD, UTRN, GRIN1, DLGAP3, DLGAP4, DLG2, HMG20A, MPRIP, GRIN2A, DLG4, DTNB, NRXN1, NLGN1, DLGAP2, KIF5A, BEGAIN, FZD4, NRXN3, SHANK2, GRIN2B, NOS1 | GO.0005623 |
| 272 | 4 | GO Function | G protein-coupled receptor binding | 0.0057 | WNT3A, FZD7, SHANK1, DLG4 | GO.0001664 |
| 968 | 7 | GO Function | protein-containing complex binding | 0.0057 | SHANK1, MLC1, DLGAP1, UTRN, GRIN1, DLG4, FZD4 | GO.0044877 |
| 109 | 3 | GO Process | memory | 0.0057 | SHANK1, GRIN1, GRIN2A | GO.0007613 |
| 128 | 3 | KEGG Pathways | Dopaminergic synapse | 0.0058 | GRIN2A, KIF5A, GRIN2B | hsa04728 |
| 251 | 4 | GO Process | positive regulation of cytosolic calcium ion concentration | 0.0058 | GRIN1, GRIN2A, DLG4, GRIN2B | GO.0007204 |
| 663 | 6 | GO Process | positive regulation of cell death | 0.0058 | GAPDH, WNT3A, ERBB4, GRIN1, GRIN2A, GRIN2B | GO.0010942 |
| 27 | 2 | Reactome Pathways | Trafficking of AMPA receptors | 0.0058 | DLG1, DLG4 | HSA-399719 |
| 27 | 2 | Reactome Pathways | Glutamate binding, activation of AMPA receptors and synaptic plasticity | 0.0058 | DLG1, DLG4 | HSA-399721 |
| 111 | 3 | GO Process | striated muscle contraction | 0.0059 | KCNJ2, DMD, NOS1 | GO.0006941 |
| 31 | 2 | GO Function | Wnt-protein binding | 0.0061 | FZD7, FZD4 | GO.0017147 |
| 44 | 2 | SMART Domains | Kinesin motor, catalytic domain. ATPase. | 0.0061 | KIF1B, KIF5A | SM00129 |
| 29 | 2 | NetworkNeighborAL | Laminin interactions, and p21 activated kinase binding domain | 0.0063 | DMD, UTRN | CL.1084 |
| 32 | 2 | GO Function | ATP-dependent microtubule motor activity | 0.0064 | KIF1B, KIF5A | GO.1990939 |
| 47 | 2 | SMART Domains | Domain with 2 conserved Trp (W) residues | 0.0064 | DMD, UTRN | SM00456 |
| 260 | 4 | GO Process | negative regulation of neurogenesis | 0.0065 | WNT3A, ERBB4, HMG20A, NLGN1 | GO.0050768 |
| 27 | 2 | GO Process | regulation of ryanodine-sensitive calcium-release channel activity | 0.0065 | DMD, NOS1 | GO.0060314 |
| 30 | 2 | Reactome Pathways | Activation of kainate receptors upon glutamate binding | 0.0065 | DLG1, DLG4 | HSA-451326 |
| 153 | 3 | Pfam | Variant SH3 domain | 0.0066 | SHANK1, SH3D19, SHANK2 | PF14604 |
| 941 | 7 | GO Process | positive regulation of protein phosphorylation | 0.0066 | WNT3A, FZD7, ERBB4, DLG1, DLG4, NRXN1, FZD4 | GO.0001934 |
| 138 | 3 | KEGG Pathways | Signaling pathways regulating pluripotency of stem cells | 0.0067 | WNT3A, FZD7, FZD4 | hsa04550 |
| 148 | 3 | KEGG Pathways | mTOR signaling pathway | 0.0069 | WNT3A, FZD7, FZD4 | hsa04150 |
| 143 | 3 | KEGG Pathways | Wnt signaling pathway | 0.0069 | WNT3A, FZD7, FZD4 | hsa04310 |
| 153 | 3 | KEGG Pathways | Cushing's syndrome | 0.0069 | WNT3A, FZD7, FZD4 | hsa04934 |
| 142 | 3 | KEGG Pathways | Alcoholism | 0.0069 | GRIN1, GRIN2A, GRIN2B | hsa05034 |
| 147 | 3 | KEGG Pathways | Breast cancer | 0.0069 | WNT3A, FZD7, FZD4 | hsa05224 |
| 147 | 3 | KEGG Pathways | Gastric cancer | 0.0069 | WNT3A, FZD7, FZD4 | hsa05226 |
| 44 | 2 | Pfam | Kinesin motor domain | 0.0069 | KIF1B, KIF5A | PF00225 |
| 43 | 2 | Pfam | Microtubule binding | 0.0069 | KIF1B, KIF5A | PF16796 |
| 1236 | 8 | GO Process | phosphorylation | 0.0069 | GAPDH, ERBB4, DLG1, GRIN1, DLG2, GRIN2A, DLG4, GRIN2B | GO.0016310 |
| 953 | 7 | GO Process | cytoskeleton organization | 0.0071 | GAPDH, SHANK1, SH3D19, DTNBP1, DLG1, DMD, NLGN1 | GO.0007010 |
| 300 | 4 | GO Component | membrane raft | 0.0072 | MLC1, DLG1, DMD, NOS1 | GO.0045121 |
| 29 | 2 | GO Process | calcium-dependent cell-cell adhesion via plasma membrane cell adhesion molecules | 0.0073 | NRXN1, NLGN1 | GO.0016339 |
| 163 | 3 | KEGG Pathways | Hepatocellular carcinoma | 0.0077 | WNT3A, FZD7, FZD4 | hsa05225 |
| 3033 | 13 | GO Process | regulation of signal transduction | 0.0079 | WNT3A, FZD7, SHANK1, DTNBP1, ERBB4, DLG1, DMD, GRIN1, DLG4, NRXN1, NLGN1, FZD4, NOS1 | GO.0009966 |
| 34 | 2 | Reactome Pathways | Signaling by WNT in cancer | 0.0079 | WNT3A, FZD4 | HSA-4791275 |
| 37 | 2 | GO Function | frizzled binding | 0.008 | WNT3A, FZD7 | GO.0005109 |
| 712 | 6 | GO Process | regulation of MAPK cascade | 0.008 | FZD7, DTNBP1, ERBB4, DLG1, NRXN1, FZD4 | GO.0043408 |
| 31 | 2 | GO Process | response to amphetamine | 0.0081 | GRIN1, GRIN2A | GO.0001975 |
| 38 | 2 | GO Function | calcium channel regulator activity | 0.0083 | NRXN2, NRXN1 | GO.0005246 |
| 3882 | 15 | GO Process | regulation of response to stimulus | 0.0083 | WNT3A, FZD7, SHANK1, MLC1, DTNBP1, ERBB4, DLG1, DMD, UTRN, GRIN1, DLG4, NRXN1, NLGN1, FZD4, NOS1 | GO.0048583 |
| 1286 | 8 | GO Process | positive regulation of developmental process | 0.0084 | WNT3A, SHANK1, ERBB4, DMD, DLG4, NRXN1, NLGN1, FZD4 | GO.0051094 |
| 32 | 2 | GO Process | regulation of membrane repolarization | 0.0084 | KCNJ2, DLG1 | GO.0060306 |
| 32 | 2 | GO Process | regulation of cardiac muscle cell differentiation | 0.0084 | WNT3A, FZD7 | GO.2000725 |
| 317 | 4 | GO Component | cell projection membrane | 0.0085 | DLG1, DMD, UTRN, SHANK2 | GO.0031253 |
| 995 | 7 | GO Process | ion transmembrane transport | 0.0085 | KCNJ2, WNT3A, DLG1, GRIN1, GRIN2A, DLG4, GRIN2B | GO.0034220 |
| 6212 | 20 | GO Process | cellular response to stimulus | 0.0085 | GAPDH, KCNJ2, TIPIN, KIF1B, NRXN2, WNT3A, FZD7, MLC1, ERBB4, DMD, GRIN1, DLG2, GRIN2A, DLG4, NRXN1, NLGN1, FZD4, NRXN3, GRIN2B, NOS1 | GO.0051716 |
| 1292 | 8 | GO Process | ion transport | 0.0086 | KCNJ2, WNT3A, MLC1, DLG1, GRIN1, GRIN2A, DLG4, GRIN2B | GO.0006811 |
| 131 | 3 | GO Process | regulation of dendrite development | 0.0086 | SHANK1, GRIN1, NLGN1 | GO.0050773 |
| 287 | 4 | GO Process | positive regulation of cellular protein localization | 0.0086 | WNT3A, ERBB4, DLG1, NRXN1 | GO.1903829 |
| 131 | 3 | GO Process | positive regulation of cation transmembrane transport | 0.0086 | KCNJ2, DMD, NOS1 | GO.1904064 |
| 40 | 2 | GO Function | kinesin binding | 0.0087 | KIF1B, KIF5A | GO.0019894 |
| 41 | 2 | InterPro Domains | Kinesin motor domain, conserved site | 0.0087 | KIF1B, KIF5A | IPR019821 |
| 152 | 3 | GO Component | endocytic vesicle membrane | 0.0089 | WNT3A, DLG4, FZD4 | GO.0030666 |
| 291 | 4 | GO Process | nucleotide biosynthetic process | 0.0089 | GAPDH, DLG1, DLG2, DLG4 | GO.0009165 |
| 36 | 2 | NetworkNeighborAL | Wnt, and Wnt-activated receptor activity | 0.009 | WNT3A, FZD7 | CL.5707 |
| 134 | 3 | GO Process | response to ethanol | 0.009 | GRIN1, GRIN2A, GRIN2B | GO.0045471 |
| 499 | 5 | GO Process | cellular metal ion homeostasis | 0.0091 | KCNJ2, GRIN1, GRIN2A, DLG4, GRIN2B | GO.0006875 |
| 34 | 2 | GO Process | long-term memory | 0.0091 | SHANK1, GRIN1 | GO.0007616 |
| 742 | 6 | GO Process | regulation of proteolysis | 0.0092 | GAPDH, WNT3A, SH3D19, GRIN1, GRIN2A, GRIN2B | GO.0030162 |
| 43 | 2 | InterPro Domains | Kinesin-like protein | 0.0093 | KIF1B, KIF5A | IPR027640 |
| 136 | 3 | GO Process | regulation of peptidyl-serine phosphorylation | 0.0093 | WNT3A, DMD, NRXN1 | GO.0033135 |
| 44 | 2 | InterPro Domains | Kinesin motor domain | 0.0094 | KIF1B, KIF5A | IPR001752 |
| 35 | 2 | GO Process | cortical cytoskeleton organization | 0.0095 | DLG1, NLGN1 | GO.0030865 |
| 35 | 2 | GO Process | synaptic vesicle recycling | 0.0095 | WNT3A, NLGN1 | GO.0036465 |
| 36 | 2 | GO Process | regulation of cardiac muscle cell contraction | 0.0099 | KCNJ2, DLG1 | GO.0086004 |
| 46 | 2 | InterPro Domains | WW domain superfamily | 0.01 | DMD, UTRN | IPR036020 |
| 39 | 2 | NetworkNeighborAL | mixed, incl. Laminin interactions, and p21 activated kinase binding domain | 0.01 | DMD, UTRN | CL.1083 |
| 512 | 5 | GO Process | protein complex oligomerization | 0.01 | KCNJ2, MLC1, GRIN1, NLGN1, GRIN2B | GO.0051259 |
| 517 | 5 | GO Process | positive regulation of protein kinase activity | 0.0104 | WNT3A, DLG1, DLG4, NRXN1, FZD4 | GO.0045860 |
| 37 | 2 | GO Process | positive regulation of dendritic spine development | 0.0104 | SHANK1, NLGN1 | GO.0060999 |
| 41 | 2 | NetworkNeighborAL | frizzled binding, and Wnt-protein binding | 0.0106 | WNT3A, FZD7 | CL.5705 |
| 45 | 2 | GO Component | T-tubule | 0.0107 | KCNJ2, NOS1 | GO.0030315 |
| 64 | 2 | SMART Domains | Calponin homology domain | 0.0107 | DMD, UTRN | SM00033 |
| 208 | 3 | SMART Domains | Epidermal growth factor-like domain. | 0.0107 | NRXN2, NRXN1, NRXN3 | SM00181 |
| 38 | 2 | GO Process | regulation of gastrulation | 0.0108 | WNT3A, FZD7 | GO.0010470 |
| 38 | 2 | GO Process | substrate adhesion-dependent cell spreading | 0.0108 | FZD7, FZD4 | GO.0034446 |
| 41 | 2 | Reactome Pathways | EPHB-mediated forward signaling | 0.0109 | GRIN1, GRIN2B | HSA-3928662 |
| 46 | 2 | GO Component | photoreceptor inner segment | 0.011 | SHANK2, NOS1 | GO.0001917 |
| 46 | 2 | GO Function | NADP binding | 0.0112 | GAPDH, NOS1 | GO.0050661 |
| 47 | 2 | GO Function | neurotransmitter binding | 0.0113 | GRIN1, GRIN2B | GO.0042165 |
| 39 | 2 | GO Process | establishment or maintenance of epithelial cell apical/basal polarity | 0.0113 | DLG1, DLG4 | GO.0045197 |
| 12432 | 30 | GO Component | organelle | 0.0115 | GAPDH, KCNJ2, TIPIN, KIF1B, WNT3A, FZD7, SHANK1, SH3D19, MLC1, DLGAP1, DTNBP1, ERBB4, DLG1, DMD, UTRN, GRIN1, DLGAP3, DLG2, HMG20A, MPRIP, GRIN2A, DLG4, NRXN1, NLGN1, DLGAP2, KIF5A, FZD4, SHANK2, GRIN2B, NOS1 | GO.0043226 |
| 51 | 2 | InterPro Domains | WW domain | 0.0118 | DMD, UTRN | IPR001202 |
| 195 | 3 | KEGG Pathways | cAMP signaling pathway | 0.0118 | GRIN1, GRIN2A, GRIN2B | hsa04024 |
| 193 | 3 | KEGG Pathways | Huntington's disease | 0.0118 | GRIN1, DLG4, GRIN2B | hsa05016 |
| 40 | 2 | GO Process | regulation of receptor internalization | 0.0118 | WNT3A, DLG4 | GO.0002090 |
| 40 | 2 | GO Process | positive regulation of potassium ion transport | 0.0118 | KCNJ2, DLG1 | GO.0043268 |
| 788 | 6 | GO Process | regulation of protein kinase activity | 0.0118 | WNT3A, DTNBP1, DLG1, DLG4, NRXN1, FZD4 | GO.0045859 |
| 323 | 4 | GO Process | response to acid chemical | 0.0121 | WNT3A, FZD7, GRIN1, FZD4 | GO.0001101 |
| 203 | 3 | KEGG Pathways | Rap1 signaling pathway | 0.0125 | GRIN1, GRIN2A, GRIN2B | hsa04015 |
| 547 | 5 | GO Process | response to wounding | 0.0127 | WNT3A, FZD7, DTNBP1, ERBB4, GRIN2A | GO.0009611 |
| 42 | 2 | GO Process | regulation of filopodium assembly | 0.0127 | NRXN1, NLGN1 | GO.0051489 |
| 42 | 2 | GO Process | positive regulation of cardiac muscle tissue development | 0.0127 | WNT3A, ERBB4 | GO.0055025 |
| 51 | 2 | GO Component | intercalated disc | 0.0129 | KCNJ2, DLG1 | GO.0014704 |
| 52 | 2 | GO Component | kinesin complex | 0.0132 | KIF1B, KIF5A | GO.0005871 |
| 9377 | 25 | GO Component | cytoplasmic part | 0.0132 | GAPDH, KCNJ2, KIF1B, WNT3A, FZD7, SHANK1, SH3D19, MLC1, DTNBP1, ERBB4, DLG1, DMD, UTRN, GRIN1, DLG2, MPRIP, GRIN2A, DLG4, NRXN1, NLGN1, DLGAP2, KIF5A, FZD4, SHANK2, NOS1 | GO.0044444 |
| 873 | 6 | GO Component | supramolecular fiber | 0.0132 | KIF1B, DLG1, DMD, DLGAP2, KIF5A, NOS1 | GO.0099512 |
| 11238 | 28 | GO Component | cytoplasm | 0.0135 | GAPDH, KCNJ2, TIPIN, KIF1B, WNT3A, FZD7, SHANK1, SH3D19, MLC1, DTNBP1, ERBB4, DLG1, DMD, UTRN, GRIN1, DLG2, MPRIP, GRIN2A, DLG4, DTNB, NRXN1, NLGN1, DLGAP2, KIF5A, BEGAIN, FZD4, SHANK2, NOS1 | GO.0005737 |
| 2226 | 10 | GO Component | cytoplasmic vesicle | 0.0135 | KIF1B, WNT3A, FZD7, MLC1, DTNBP1, GRIN1, GRIN2A, DLG4, NRXN1, FZD4 | GO.0031410 |
| 45 | 2 | GO Process | visual learning | 0.0142 | GRIN1, GRIN2A | GO.0008542 |
| 1427 | 8 | GO Process | response to oxygen-containing compound | 0.0142 | KIF1B, WNT3A, FZD7, MLC1, GRIN1, GRIN2A, FZD4, GRIN2B | GO.1901700 |
| 1764 | 9 | GO Process | regulation of intracellular signal transduction | 0.0142 | FZD7, DTNBP1, ERBB4, DLG1, DMD, NRXN1, NLGN1, FZD4, NOS1 | GO.1902531 |
| 57 | 2 | GO Component | neuromuscular junction | 0.0144 | DLG1, UTRN | GO.0031594 |
| 32 | 2 | UniProt Keywords | Amyotrophic lateral sclerosis | 0.0146 | ERBB4, KIF5A | KW-0036 |
| 67 | 2 | Pfam | Calponin homology (CH) domain | 0.0147 | DMD, UTRN | PF00307 |
| 46 | 2 | GO Process | cardiac muscle cell action potential | 0.0147 | KCNJ2, DMD | GO.0086001 |
| 59 | 2 | InterPro Domains | GPCR, family 2-like | 0.0151 | FZD7, FZD4 | IPR017981 |
| 47 | 2 | GO Process | regulation of sodium ion transmembrane transporter activity | 0.0152 | DMD, UTRN | GO.2000649 |
| 577 | 5 | GO Process | regulation of signaling receptor activity | 0.0154 | WNT3A, SHANK1, DLG4, NRXN1, NLGN1 | GO.0010469 |
| 169 | 3 | GO Process | central nervous system neuron differentiation | 0.0154 | WNT3A, ERBB4, NRXN1 | GO.0021953 |
| 169 | 3 | GO Process | regulation of JNK cascade | 0.0154 | FZD7, DTNBP1, FZD4 | GO.0046328 |
| 48 | 2 | GO Process | regulation of action potential | 0.0156 | KCNJ2, DLG1 | GO.0098900 |
| 194 | 3 | GO Function | protein C-terminus binding | 0.0158 | SHANK1, DLG1, DLG4 | GO.0008022 |
| 1144 | 7 | GO Process | locomotion | 0.0161 | WNT3A, ERBB4, GRIN2A, NRXN1, KIF5A, FZD4, NRXN3 | GO.0040011 |
| 402 | 4 | GO Component | cell-cell junction | 0.0162 | KCNJ2, MLC1, DLG1, FZD4 | GO.0005911 |
| 1223 | 7 | GO Function | transporter activity | 0.0163 | KCNJ2, MLC1, DLG1, GRIN1, GRIN2A, DLG4, GRIN2B | GO.0005215 |
| 186 | 3 | NetworkNeighborAL | Wnt signaling pathway, and TGF-beta signaling pathway | 0.0163 | WNT3A, FZD7, FZD4 | CL.5698 |
| 55 | 2 | NetworkNeighborAL | frizzled binding, and Signaling by WNT in cancer | 0.0163 | WNT3A, FZD7 | CL.5703 |
| 86 | 2 | SMART Domains | Sterile alpha motif. | 0.0165 | SHANK1, SHANK2 | SM00454 |
| 228 | 3 | KEGG Pathways | Ras signaling pathway | 0.0166 | GRIN1, GRIN2A, GRIN2B | hsa04014 |
| 50 | 2 | GO Process | response to bronchodilator | 0.0167 | GRIN1, GRIN2A | GO.0097366 |
| 1826 | 9 | GO Process | positive regulation of gene expression | 0.0172 | KIF1B, WNT3A, FZD7, DTNBP1, ERBB4, GRIN1, NRXN1, FZD4, NOS1 | GO.0010628 |
| 51 | 2 | GO Process | Wnt signaling pathway, planar cell polarity pathway | 0.0172 | FZD7, FZD4 | GO.0060071 |
| 612 | 5 | Reactome Pathways | Membrane Trafficking | 0.0176 | KIF1B, SH3D19, DTNBP1, KIF5A, FZD4 | HSA-199991 |
| 54 | 2 | Reactome Pathways | Golgi Associated Vesicle Biogenesis | 0.0176 | SH3D19, DTNBP1 | HSA-432722 |
| 180 | 3 | GO Process | positive regulation of peptidyl-tyrosine phosphorylation | 0.0179 | WNT3A, ERBB4, DLG4 | GO.0050731 |
| 604 | 5 | GO Process | positive regulation of apoptotic process | 0.0181 | GAPDH, WNT3A, ERBB4, GRIN1, GRIN2A | GO.0043065 |
| 670 | 5 | GO Component | perinuclear region of cytoplasm | 0.0183 | GAPDH, MLC1, DLG1, KIF5A, NOS1 | GO.0048471 |
| 182 | 3 | GO Process | striated muscle cell differentiation | 0.0183 | WNT3A, DMD, NOS1 | GO.0051146 |
| 64 | 2 | GO Function | phosphatidylinositol-4,5-bisphosphate binding | 0.0185 | KCNJ2, FZD7 | GO.0005546 |
| 54 | 2 | GO Process | heterophilic cell-cell adhesion via plasma membrane cell adhesion molecules | 0.0188 | NRXN1, NLGN1 | GO.0007157 |
| 184 | 3 | GO Process | locomotory behavior | 0.0188 | GRIN1, DLG4, FZD4 | GO.0007626 |
| 54 | 2 | GO Process | tissue regeneration | 0.0188 | FZD7, ERBB4 | GO.0042246 |
| 58 | 2 | Reactome Pathways | Non-integrin membrane-ECM interactions | 0.0188 | DMD, NRXN1 | HSA-3000171 |
| 198 | 3 | Reactome Pathways | Muscle contraction | 0.0188 | KCNJ2, DMD, NOS1 | HSA-397014 |
| 61 | 2 | Reactome Pathways | Asymmetric localization of PCP proteins | 0.0188 | FZD7, FZD4 | HSA-4608870 |
| 649 | 5 | Reactome Pathways | Vesicle-mediated transport | 0.0188 | KIF1B, SH3D19, DTNBP1, KIF5A, FZD4 | HSA-5653656 |
| 58 | 2 | Reactome Pathways | Transcriptional Regulation by MECP2 | 0.0188 | GRIN2A, GRIN2B | HSA-8986944 |
| 58 | 2 | Reactome Pathways | Kinesins | 0.0188 | KIF1B, KIF5A | HSA-983189 |
| 63 | 2 | NetworkNeighborAL | COPI-dependent Golgi-to-ER retrograde traffic | 0.0192 | KIF1B, KIF5A | CL.18499 |
| 63 | 2 | NetworkNeighborAL | mixed, incl. dystrophin-associated glycoprotein complex, and Annexin | 0.0192 | DTNB, NOS1 | CL.2055 |
| 615 | 5 | GO Process | tube morphogenesis | 0.0192 | WNT3A, DLG1, NRXN1, FZD4, NRXN3 | GO.0035239 |
| 55 | 2 | GO Process | negative regulation of cell-substrate adhesion | 0.0193 | FZD7, FZD4 | GO.0010812 |
| 618 | 5 | GO Process | inorganic cation transmembrane transport | 0.0194 | KCNJ2, WNT3A, GRIN1, GRIN2A, GRIN2B | GO.0098662 |
| 81 | 2 | Pfam | EGF-like domain | 0.0198 | NRXN1, NRXN3 | PF00008 |
| 83 | 2 | Pfam | SAM domain (Sterile alpha motif) | 0.0198 | SHANK1, SHANK2 | PF00536 |
| 80 | 2 | Pfam | SAM domain (Sterile alpha motif) | 0.0198 | SHANK1, SHANK2 | PF07647 |
| 189 | 3 | GO Process | regulation of cell-substrate adhesion | 0.0198 | FZD7, UTRN, FZD4 | GO.0010810 |
| 70 | 2 | GO Component | sarcoplasm | 0.02 | DTNBP1, NOS1 | GO.0016528 |
| 623 | 5 | GO Process | regulation of cell adhesion | 0.02 | WNT3A, FZD7, DLG1, UTRN, FZD4 | GO.0030155 |
| 220 | 3 | InterPro Domains | Concanavalin A-like lectin/glucanase domain superfamily | 0.0201 | NRXN2, NRXN1, NRXN3 | IPR013320 |
| 900 | 6 | GO Process | response to drug | 0.0202 | KIF1B, GRIN1, GRIN2A, FZD4, GRIN2B, NOS1 | GO.0042493 |
| 901 | 6 | GO Process | regulation of protein localization | 0.0203 | GAPDH, WNT3A, ERBB4, DLG1, NRXN1, NLGN1 | GO.0032880 |
| 678 | 5 | GO Function | kinase binding | 0.0204 | KIF1B, DLG1, UTRN, DLG2, DLG4 | GO.0019900 |
| 225 | 3 | InterPro Domains | EGF-like domain | 0.0209 | NRXN2, NRXN1, NRXN3 | IPR000742 |
| 196 | 3 | GO Process | cellular response to acid chemical | 0.0214 | WNT3A, FZD7, FZD4 | GO.0071229 |
| 59 | 2 | GO Process | regulation of postsynapse organization | 0.0214 | NLGN1, GRIN2B | GO.0099175 |
| 74 | 2 | GO Component | caveola | 0.0219 | MLC1, NOS1 | GO.0005901 |
| 60 | 2 | GO Process | positive regulation of protein tyrosine kinase activity | 0.022 | WNT3A, DLG4 | GO.0061098 |
| 75 | 2 | GO Component | presynaptic membrane | 0.0223 | GRIN2A, NRXN1 | GO.0042734 |
| 1238 | 7 | GO Process | regulation of hydrolase activity | 0.0228 | GAPDH, WNT3A, GRIN1, DLG2, GRIN2A, GRIN2B, NOS1 | GO.0051336 |
| 77 | 2 | GO Component | cortical cytoskeleton | 0.0232 | UTRN, DLG4 | GO.0030863 |
| 203 | 3 | GO Process | regulation of protein kinase B signaling | 0.0232 | ERBB4, DLG1, NRXN1 | GO.0051896 |
| 72 | 2 | Reactome Pathways | trans-Golgi Network Vesicle Budding | 0.0236 | SH3D19, DTNBP1 | HSA-199992 |
| 72 | 2 | Reactome Pathways | Clathrin derived vesicle budding | 0.0236 | SH3D19, DTNBP1 | HSA-421837 |
| 724 | 5 | GO Component | cytoplasmic vesicle membrane | 0.0237 | KIF1B, WNT3A, DTNBP1, DLG4, FZD4 | GO.0030659 |
| 460 | 4 | GO Component | neuronal cell body | 0.0237 | KCNJ2, NRXN1, KIF5A, SHANK2 | GO.0043025 |
| 63 | 2 | GO Process | forebrain generation of neurons | 0.0237 | WNT3A, ERBB4 | GO.0021872 |
| 205 | 3 | GO Process | negative regulation of neuron differentiation | 0.0237 | WNT3A, HMG20A, NLGN1 | GO.0045665 |
| 79 | 2 | InterPro Domains | Kinesin motor domain superfamily | 0.0242 | KIF1B, KIF5A | IPR036961 |
| 207 | 3 | GO Process | regulation of animal organ morphogenesis | 0.0242 | WNT3A, FZD7, FZD4 | GO.2000027 |
| 3131 | 12 | GO Process | organelle organization | 0.0248 | GAPDH, NRXN2, SHANK1, SH3D19, DTNBP1, ERBB4, DLG1, DMD, HMG20A, DLG4, NRXN1, NLGN1 | GO.0006996 |
| 65 | 2 | GO Process | cellular response to calcium ion | 0.0248 | NRXN1, NLGN1 | GO.0071277 |
| 82 | 2 | InterPro Domains | Calponin homology domain | 0.0253 | DMD, UTRN | IPR001715 |
| 272 | 3 | KEGG Pathways | Neuroactive ligand-receptor interaction | 0.0254 | GRIN1, GRIN2A, GRIN2B | hsa04080 |
| 94 | 2 | KEGG Pathways | Systemic lupus erythematosus | 0.0254 | GRIN2A, GRIN2B | hsa05322 |
| 959 | 6 | GO Process | positive regulation of intracellular signal transduction | 0.0258 | FZD7, ERBB4, NRXN1, NLGN1, FZD4, NOS1 | GO.1902533 |
| 84 | 2 | InterPro Domains | CH domain superfamily | 0.0259 | DMD, UTRN | IPR036872 |
| 458 | 4 | GO Function | metal ion transmembrane transporter activity | 0.0261 | KCNJ2, GRIN1, GRIN2A, GRIN2B | GO.0046873 |
| 425 | 4 | GO Process | purine ribonucleotide metabolic process | 0.0267 | GAPDH, DLG1, DLG2, DLG4 | GO.0009150 |
| 683 | 5 | GO Process | negative regulation of cell differentiation | 0.0274 | WNT3A, FZD7, ERBB4, HMG20A, NLGN1 | GO.0045596 |
| 261 | 3 | InterPro Domains | EF-hand domain pair | 0.0282 | DMD, UTRN, DTNB | IPR011992 |
| 91 | 2 | InterPro Domains | Sterile alpha motif domain | 0.0287 | SHANK1, SHANK2 | IPR001660 |
| 1051 | 6 | GO Function | transition metal ion binding | 0.0293 | DMD, UTRN, GRIN2A, DTNB, GRIN2B, NOS1 | GO.0046914 |
| 253 | 3 | GO Function | microtubule binding | 0.0298 | GAPDH, KIF1B, KIF5A | GO.0008017 |
| 73 | 2 | GO Process | cardiac muscle contraction | 0.0301 | KCNJ2, DMD | GO.0060048 |
| 73 | 2 | GO Process | regulation of potassium ion transmembrane transport | 0.0301 | KCNJ2, DLG1 | GO.1901379 |
| 2815 | 11 | GO Process | response to organic substance | 0.0306 | GAPDH, KIF1B, WNT3A, FZD7, MLC1, ERBB4, GRIN1, GRIN2A, FZD4, GRIN2B, NOS1 | GO.0010033 |
| 229 | 3 | GO Process | regulation of endocytosis | 0.0306 | WNT3A, DLG4, NLGN1 | GO.0030100 |
| 6516 | 19 | GO Process | regulation of metabolic process | 0.0317 | GAPDH, TIPIN, KIF1B, WNT3A, FZD7, SH3D19, DTNBP1, ERBB4, DLG1, DMD, GRIN1, DLG2, HMG20A, GRIN2A, DLG4, NRXN1, FZD4, GRIN2B, NOS1 | GO.0019222 |
| 86 | 2 | NetworkNeighborAL | mixed, incl. ECM-receptor interaction, and cell-substrate junction assembly | 0.0323 | DMD, UTRN | CL.989 |
| 77 | 2 | GO Process | regulation of dendrite morphogenesis | 0.0326 | GRIN1, NLGN1 | GO.0048814 |
| 237 | 3 | GO Process | telencephalon development | 0.0328 | WNT3A, ERBB4, GRIN1 | GO.0021537 |
| 457 | 4 | GO Process | muscle structure development | 0.0328 | WNT3A, DMD, UTRN, NOS1 | GO.0061061 |
| 89 | 2 | Reactome Pathways | PCP/CE pathway | 0.0332 | FZD7, FZD4 | HSA-4086400 |
| 100 | 2 | InterPro Domains | EGF-type aspartate/asparagine hydroxylation site | 0.0335 | NRXN1, NRXN3 | IPR000152 |
| 461 | 4 | GO Process | wound healing | 0.0336 | WNT3A, FZD7, DTNBP1, ERBB4 | GO.0042060 |
| 79 | 2 | GO Process | ephrin receptor signaling pathway | 0.0337 | GRIN1, GRIN2B | GO.0048013 |
| 91 | 2 | Reactome Pathways | EPH-Ephrin signaling | 0.0338 | GRIN1, GRIN2B | HSA-2682334 |
| 6072 | 18 | GO Process | regulation of macromolecule metabolic process | 0.0346 | GAPDH, TIPIN, KIF1B, WNT3A, FZD7, SH3D19, DTNBP1, ERBB4, DLG1, DMD, GRIN1, HMG20A, GRIN2A, DLG4, NRXN1, FZD4, GRIN2B, NOS1 | GO.0060255 |
| 91 | 2 | NetworkNeighborAL | mixed, incl. ECM-receptor interaction, and Actinin-type actin-binding domain, conserved site | 0.0349 | DMD, UTRN | CL.988 |
| 245 | 3 | GO Process | negative regulation of cell adhesion | 0.0351 | FZD7, DLG1, FZD4 | GO.0007162 |
| 468 | 4 | GO Process | response to toxic substance | 0.0351 | KIF1B, GRIN1, GRIN2A, GRIN2B | GO.0009636 |
| 6082 | 18 | GO Process | regulation of cellular metabolic process | 0.0351 | GAPDH, TIPIN, WNT3A, FZD7, SH3D19, DTNBP1, ERBB4, DLG1, DMD, GRIN1, DLG2, HMG20A, GRIN2A, DLG4, NRXN1, FZD4, GRIN2B, NOS1 | GO.0031323 |
| 83 | 2 | GO Process | negative regulation of cell morphogenesis involved in differentiation | 0.0364 | WNT3A, NLGN1 | GO.0010771 |
| 96 | 2 | Reactome Pathways | COPI-dependent Golgi-to-ER retrograde traffic | 0.0364 | KIF1B, KIF5A | HSA-6811434 |
| 2605 | 10 | Reactome Pathways | Signal Transduction | 0.0365 | WNT3A, FZD7, DLG1, GRIN1, DLG2, GRIN2A, DLG4, KIF5A, FZD4, GRIN2B | HSA-162582 |
| 800 | 5 | GO Function | zinc ion binding | 0.0366 | DMD, UTRN, GRIN2A, DTNB, GRIN2B | GO.0008270 |
| 11116 | 27 | GO Process | regulation of biological process | 0.0371 | GAPDH, KCNJ2, TIPIN, KIF1B, NRXN2, WNT3A, FZD7, SHANK1, SH3D19, MLC1, DTNBP1, ERBB4, DLG1, DMD, UTRN, GRIN1, DLG2, HMG20A, GRIN2A, DLG4, NRXN1, NLGN1, FZD4, NRXN3, SHANK2, GRIN2B, NOS1 | GO.0050789 |
| 84 | 2 | GO Process | actin-mediated cell contraction | 0.0371 | KCNJ2, DMD | GO.0070252 |
| 55 | 2 | UniProt Keywords | S-nitrosylation | 0.0375 | GAPDH, KCNJ2 | KW-0702 |
| 191 | 3 | UniProt Keywords | Wnt signaling pathway | 0.0375 | WNT3A, FZD7, FZD4 | KW-0879 |
| 187 | 3 | UniProt Keywords | Epilepsy | 0.0375 | GRIN2A, KIF5A, GRIN2B | KW-0887 |
| 254 | 3 | GO Process | regulation of cellular response to growth factor stimulus | 0.0383 | DMD, NRXN1, FZD4 | GO.0090287 |
| 86 | 2 | GO Process | regulation of JUN kinase activity | 0.0385 | DTNBP1, FZD4 | GO.0043506 |
| 2926 | 11 | GO Process | animal organ development | 0.0386 | WNT3A, FZD7, ERBB4, DLG1, DMD, UTRN, GRIN1, GRIN2A, NRXN1, FZD4, GRIN2B | GO.0048513 |
| 486 | 4 | GO Process | cellular response to lipid | 0.0389 | WNT3A, FZD7, MLC1, FZD4 | GO.0071396 |
| 849 | 5 | InterPro Domains | P-loop containing nucleoside triphosphate hydrolase | 0.0391 | KIF1B, DLG1, DLG2, DLG4, KIF5A | IPR027417 |
| 87 | 2 | GO Process | neurotransmitter metabolic process | 0.0391 | GRIN2A, NOS1 | GO.0042133 |
| 1075 | 6 | GO Process | negative regulation of protein metabolic process | 0.0401 | GAPDH, DTNBP1, DLG1, DMD, HMG20A, GRIN2A | GO.0051248 |
| 113 | 2 | InterPro Domains | Sterile alpha motif/pointed domain superfamily | 0.0403 | SHANK1, SHANK2 | IPR013761 |
| 493 | 4 | GO Process | actin filament-based process | 0.0403 | KCNJ2, DTNBP1, DLG1, DMD | GO.0030029 |
| 89 | 2 | GO Process | synaptic vesicle transport | 0.0403 | DTNBP1, NLGN1 | GO.0048489 |
| 89 | 2 | GO Process | establishment of synaptic vesicle localization | 0.0403 | DTNBP1, NLGN1 | GO.0097480 |
| 10223 | 26 | UniProt Keywords | Alternative splicing | 0.0409 | GAPDH, KIF1B, NRXN2, WNT3A, SHANK1, SH3D19, MLC1, DLGAP1, DTNBP1, ERBB4, DLG1, DMD, GRIN1, DLGAP4, DLG2, HMG20A, MPRIP, GRIN2A, DLG4, DTNB, NRXN1, NLGN1, DLGAP2, NRXN3, SHANK2, NOS1 | KW-0025 |
| 263 | 3 | GO Process | regulation of cell morphogenesis involved in differentiation | 0.0409 | WNT3A, GRIN1, NLGN1 | GO.0010769 |
| 293 | 3 | Reactome Pathways | Signaling by WNT | 0.0409 | WNT3A, FZD7, FZD4 | HSA-195721 |
| 541 | 4 | Reactome Pathways | Axon guidance | 0.0409 | DLG1, GRIN1, DLG4, GRIN2B | HSA-422475 |
| 102 | 2 | NetworkNeighborAL | Golgi-to-ER retrograde transport, and MHC class II protein complex | 0.0419 | KIF1B, KIF5A | CL.18498 |
| 104 | 2 | NetworkNeighborAL | Focal adhesion, and cell-substrate junction assembly | 0.0422 | DMD, UTRN | CL.987 |
| 4005 | 13 | GO Component | intracellular non-membrane-bounded organelle | 0.0432 | GAPDH, TIPIN, KIF1B, DTNBP1, DLG1, DMD, UTRN, MPRIP, DLG4, NRXN1, DLGAP2, KIF5A, NOS1 | GO.0043232 |
| 307 | 3 | GO Component | apical plasma membrane | 0.0434 | MLC1, DLG1, SHANK2 | GO.0016324 |
| 4972 | 16 | UniProt Keywords | Cytoplasm | 0.0438 | GAPDH, TIPIN, KIF1B, SHANK1, SH3D19, MLC1, DTNBP1, DLG1, DMD, UTRN, MPRIP, DLG4, DTNB, KIF5A, BEGAIN, SHANK2 | KW-0963 |
| 109 | 2 | NetworkNeighborAL | Focal adhesion, and cell-substrate junction assembly | 0.0448 | DMD, UTRN | CL.986 |
| 95 | 2 | GO Process | regulation of heart rate | 0.0449 | KCNJ2, DMD | GO.0002027 |
| 512 | 4 | GO Process | positive regulation of MAPK cascade | 0.0449 | FZD7, ERBB4, NRXN1, FZD4 | GO.0043410 |
| 274 | 3 | GO Process | regulation of ERK1 and ERK2 cascade | 0.045 | ERBB4, DLG1, NRXN1 | GO.0070372 |
| 96 | 2 | GO Process | cerebellum development | 0.0453 | NRXN1, FZD4 | GO.0021549 |
| 229 | 3 | UniProt Keywords | EGF-like domain | 0.0458 | NRXN2, NRXN1, NRXN3 | KW-0245 |
| 116 | 2 | Reactome Pathways | L1CAM interactions | 0.047 | DLG1, DLG4 | HSA-373760 |
| 281 | 3 | GO Process | negative regulation of response to external stimulus | 0.0477 | WNT3A, GRIN1, NRXN1 | GO.0032102 |
| 807 | 5 | GO Process | circulatory system development | 0.0477 | WNT3A, ERBB4, NRXN1, FZD4, NRXN3 | GO.0072359 |
| 2222 | 9 | GO Process | multi-organism process | 0.0484 | GAPDH, NRXN2, SHANK1, DLG1, GRIN1, DLG4, NRXN1, NRXN3, SHANK2 | GO.0051704 |
| 120 | 2 | GO Component | cell cortex part | 0.0488 | UTRN, DLG4 | GO.0044448 |
| 122 | 2 | GO Component | Z disc | 0.0498 | DMD, NOS1 | GO.0030018 |
